# Integrated multi-omics analysis and functional experiments reveals PPAP2C as a potential prognostic biomarker and therapeutic target in breast cancer

**DOI:** 10.3389/fphar.2026.1884232

**Published:** 2026-08-24

**Authors:** Xiaoxi Lin, Lihang Cen, Sihui Yue, Liangsen Teng, Shuang Qin, Yipeng Cheng, Mingyue Qiu, Yonggang Lu, Jie Zhang, Shuguang Zuo

**Affiliations:** 1 Chifeng Municipal Hospital, Chifeng Clinical Medical School, Inner Mongolia Medical University, Chifeng, Inner Mongolia, China; 2 Liuzhou Key Laboratory of Cancer Biotherapy, Liuzhou Municipal Liutie Central Hospital, Affiliated Liuzhou Hospital of Youjiang Medical University for Nationalities, Liuzhou, Guangxi, China

**Keywords:** breast cancer, PLPP2, PPAP2C, prognostic biomarker, therapeutic target

## Abstract

**Background:**

This study aims to systematically elucidate the clinical significance and biological function of the phospholipid phosphatase (PLPP) family member (PPAP2C) phosphatidic acid phosphatase type 2C in breast cancer, and to evaluate its potential as a prognostic biomarker and therapeutic target.

**Methods:**

Gene expression data from The Cancer Genome Atlas (TCGA), Genotype-Tissue Expression (GTEx), and Cancer Cell Line Encyclopedia (CCLE) databases were integrated to characterize the expression profile of PLPP family members, focusing on PPAP2C in breast cancer. The prognostic value of PPAP2C, initially identified at the mRNA level (TCGA, (METABRIC) Molecular Taxonomy of Breast Cancer International Consortium, Gene Expression Omnibus (GEO)), was confirmed at the protein level by immunohistochemistry (IHC) on tissue microarrays (TMA). The oncogenic functions of PPAP2C were investigated in triple-negative breast cancer (TNBC) cells through CRISPR-Cas9-mediated knockout and ectopic overexpression, with assessment of key phenotypes including proliferation, colony formation, migration, and invasion. *In vivo* validation was subsequently performed using an MDA-MB-231 xenograft model.

**Results:**

PPAP2C exhibits the most significant overexpression pattern across 33 cancer types (upregulated in 16 cancers, downregulated in only 3). Compared with normal tissues, PPAP2C showed specific overexpression in breast cancer tissues and was significantly associated with advanced clinical stages and aggressive subtypes (HER2^+^ and TNBC). Survival analysis demonstrated that high PPAP2C expression correlated with significantly shorter overall survival and disease-free survival, which was further validated in METABRIC and GEO cohorts. Tissue microarray analysis confirmed higher PPAP2C protein positivity in tumor tissues (94.7%) than in adjacent normal tissues (59.7%), with worse OS and RFS in high-expression groups. Multivariate analysis identified PPAP2C as an independent prognostic factor for OS. Functional experiments revealed that PPAP2C knockout (via 5-bp/1-bp frameshift mutations) suppressed TNBC cell proliferation, colony formation, migration, and invasion, while overexpression enhanced these phenotypes. *In vivo* studies further demonstrated complete tumor regression in MDA-MB-231 xenografts upon PPAP2C knockout.

**Conclusion:**

This study identifies PPAP2C as a key oncogenic driver and a robust independent prognostic biomarker in breast cancer. The findings provide compelling evidence that PPAP2C represents a promising therapeutic target, offering a new strategic avenue for precision therapy, particularly for aggressive breast cancer subtypes.

## 1 Background

Phospholipid phosphate phosphatases (PLPPs), alternatively termed lipid phosphate phosphatases (LPPs), constitute a specialized subgroup within the integral membrane glycoprotein superfamily (Tang et al., 2015). These enzymes are characterized by a conserved structural architecture featuring six transmembrane α-helical domains and three canonical phosphatase active motifs that are evolutionarily conserved across species (Tang and Brindley, 2020). The mammalian PLPP family comprises seven distinct isoforms, systematically designated as PLPP1 through PLPP7 (with corresponding LPP1-LPP7 nomenclature). These isoforms are encoded by seven independent genes distributed throughout the genome: PPAP2A (PLPP1), PPAP2C (PLPP2), PPAP2B (PLPP3), PPAPDC1A (PLPP4), PPAPDC1B (PLPP5), PPAPDC2 (PLPP6), and PPAPDC3 (PLPP7) (Tang and Brindley, 2020).

PLPPs exhibit distinct subcellular distribution patterns, with predominant localization to both the plasma membrane and endomembrane compartments, including the endoplasmic reticulum and Golgi apparatus (Brindley et al., 2009). At these sites, they exert their catalytic activity by mediating the dephosphorylation of diverse lipid phosphoesters, operating on both extracellular and intracellular substrates. The extracellular enzymatic activity of PLPP1 and PLPP3 is particularly noteworthy, as these isoforms specifically hydrolyze two key bioactive lipids: lysophosphatidic acid (LPA) and sphingosine 1-phosphate (S1P). This hydrolysis yields monoacylglycerol (MAG) and sphingosine as respective products, thereby serving as a critical regulatory mechanism for controlling extracellular concentrations of LPA and S1P and modulating their downstream signaling networks (Albers et al., 2010; Ren et al., 2013). These lipid mediators function as potent regulators of multiple oncogenic processes, prominently featuring cellular proliferation and migratory capacity (Tang and Brindley, 2020; Xu, 2019). A compelling demonstration of this regulatory mechanism comes from studies in rat fibroblasts, where PLPP1 overexpression was shown to significantly enhance extracellular LPA dephosphorylation. This enzymatic activity results in the attenuation of LPA-stimulated mitogen-activated protein kinase (MAPK) pathway activation, ultimately leading to inhibition of cellular migration (Long et al., 2006). Beyond their extracellular functions, PLPPs also play crucial roles in intracellular lipid metabolism. They catalyze the conversion of phosphatidic acid (PA) to diacylglycerol (DAG), a transformation with profound biological implications (Nanjundan and Possmayer, 2003). As a central secondary messenger, DAG activates multiple signaling cascades, most notably the protein kinase C (PKC) pathway, thereby exerting broad influence over fundamental cellular processes including proliferation, differentiation, programmed cell death, and blood vessel formation (Kang, 2014).

Accumulating evidence suggests that aberrant PLPP expression is closely associated with tumorigenesis and progression. In breast, thyroid, and ovarian carcinomas, PLPP1 upregulation enhances extracellular LPA hydrolysis, resulting in three key anti-tumor effects: suppressed proliferation, impaired clonogenic potential, and increased apoptotic cell death (Tang et al., 2014; Tanyi et al., 2003a). This tumor-suppressive activity is further substantiated by xenograft studies showing that PLPP1-overexpressing breast cancer cells generate significantly smaller tumors with reduced metastatic capacity compared to cells expressing either catalytically inactive PLPP1 variants or GFP controls (Boucharaba et al., 2006; Tang et al., 2014). Parallel investigations demonstrate that PLPP3 exerts comparable tumor-suppressing effects in ovarian cancer models, where its overexpression leads to: (1) inhibited proliferation, (2) decreased cell viability, (3) enhanced apoptosis, and (4) suppressed tumor-forming capacity (Tanyi et al., 2003b). Conversely, PLPP4 displays a tumor-specific expression pattern, with minimal detection in normal lung epithelium but marked upregulation in NSCLC. RNAi-mediated PLPP4 silencing produces significant anti-tumor effects, including: (1) impaired proliferation, (2) cell cycle arrest, and (3) reduced tumor formation *in vivo* (Zhang et al., 2017). Similarly, PPAP2C shows consistent overexpression in multiple malignancies (lung, bladder, ovarian, prostate) relative to corresponding normal tissues (Flanagan et al., 2009). Functional analyses reveal PPAP2C’s critical role in cell cycle regulation, with knockdown experiments demonstrating delayed S-phase transition and decreased cyclin A expression, while overexpression produces accelerated S-phase progression and elevated cyclin A levels (Morris et al., 2006). These collective observations establish a functional dichotomy within the PLPP family: while PLPP1 and PLPP3 primarily function as tumor suppressors, PPAP2C and PLPP4 appear to act as oncogenic drivers in various cancer contexts.

Breast cancer configure a major global health challenge for women, characterized by persistently rising incidence rates and a concerning shift toward earlier age of onset (Siegel et al., 2021). Contemporary research in cancer metabolism has identified dysregulated phospholipid metabolism as a fundamental characteristic of breast cancer pathogenesis (Wang et al., 2024). Breast cancer cells systematically reprogram their lipid metabolic networks, with particular emphasis on phospholipid pathways, to support their aggressive proliferative capacity, invasive potential, and metastatic dissemination through both bioenergetic and signaling mechanisms (Wan et al., 2025). This metabolic rewiring involves not only the disruption of lipid anabolic-catabolic homeostasis but also the profound modification of lipid-mediated signaling networks, collectively driving the acquisition of malignant phenotypes (Huang et al., 2025). The phospholipid phosphatase (PLPP) family has emerged as a crucial regulatory node within this metabolic framework, although the comprehensive characterization of its expression profiles and clinical relevance in breast cancer remains an active area of investigation.

Recent pan-cancer studies employing integrated multi-omics approaches have successfully identified novel oncogenic drivers and prognostic biomarkers across multiple malignancies, including breast cancer (Gou et al., 2025; Wang et al., 2026). However, the functional and clinical significance of the PLPP family member PPAP2C in breast cancer has not been systematically characterized through such a comprehensive approach. Therefore, in this study, we aimed to systematically characterize the expression profile, clinical significance, and biological function of PPAP2C in breast cancer through integrated multi-omics analysis and functional experiments.

## 2 Methods

### 2.1 Data acquisition

Data were obtained and preprocessed as follows: RNA-seq data and corresponding clinical survival information for breast cancer and normal tissue samples from The Cancer Genome Atlas (TCGA) database, along with RNA-seq data of normal breast tissue from the Genotype-Tissue Expression (GTEx) database, were downloaded using the UCSC Xena browser (https://xenabrowser.net). Breast cancer RNA-seq data and patient survival information from the METABRIC dataset were acquired via cBioPortal (https://www.cbioportal.org). mRNA expression profiles and corresponding survival data from breast cancer gene microarray datasets (GSE20685 and GSE42568) were retrieved from the Gene Expression Omnibus (GEO) database (https://www.ncbi.nlm.nih.gov/geo). Additionally, mRNA expression profiles of breast cancer cell lines were obtained from the Cancer Cell Line Encyclopedia (CCLE) (https://portals.broadinstitute.org/ccle). All datasets were uniformly normalized using the transcripts per million (TPM) method.

### 2.2 Differential expression analysis

The RNA-seq data of PLPP family members were obtained from the TCGA database, encompassing 33 different cancer types. Differential expression analysis was performed using the GEPIA online tool (http://gepia2.cancer-pku.cn), employing the LIMMA (Linear Models for Microarray Data) method. The threshold for significant differential expression was set at a fold change >2 (log2FC > 1) with a p-value <0.01.

### 2.3 Gene Co-expression network analysis

Gene co-expression networks were constructed to investigate the interactions among PLPP family members in breast cancer. RNA-seq data from breast cancer tissues in the TCGA database and normal breast tissues in the GTEx database were utilized for this analysis. Spearman correlation analysis was performed to evaluate pairwise expression relationships between PLPP family members. A correlation coefficient (R) threshold of >0.3 and a statistical significance threshold of p < 0.05 were applied to identify significant co-expression relationships.

### 2.4 Analysis of correlation between gene Expression and copy number variation

The relationship between mRNA expression levels and copy number variations (CNVs) of PLPP family members was investigated. Genetic alteration data, including gene amplifications and deep deletions, were first extracted from the TCGA breast cancer dataset using the cBioPortal platform. Subsequently, Spearman correlation analysis was employed to assess the association between gene expression levels and copy number variations. This analysis was performed separately in both the TCGA breast cancer cohort and the CCLE dataset to ensure robustness. A statistically significant correlation was defined by a Spearman correlation coefficient (R) greater than 0.3 with a p-value less than 0.05.

### 2.5 DNA methylation analysis

To investigate the promoter methylation status of PPAP2C in breast cancer, we analyzed its methylation levels using the UALCAN portal (https://ualcan.path.uab.edu). Methylation beta values for PPAP2C were extracted from the TCGA breast cancer dataset, comparing primary tumor tissues with normal breast tissues. The beta values, ranging from 0 (unmethylated) to 1 (fully methylated), represent the median methylation level across all promoter CpG sites of the gene. To further assess the relationship between PPAP2C promoter methylation and its expression, we downloaded the corresponding RNA-seq expression data (log2(TPM+1) and methylation beta values for PPAP2C from the TCGA database. Spearman’s rank correlation coefficient between PPAP2C expression and its promoter methylation levels was calculated using R software, with |R| > 0.3 and P < 0.05 considered statistically significant. Results were visualized using the “ggplot2” package.

### 2.6 Analysis of correlation between PPAP2C expression and the expression of 41 known breast cancer driver genes

To evaluate the correlation between PPAP2C expression and the expression of 41 known breast cancer driver genes, we used the RNA-seq expression data obtained from the TCGA database as described in Section 2.5. Spearman’s rank correlation coefficients between PPAP2C and the 41 driver genes (Kim et al., 2026) were calculated using the “Ggally” package in R. Correlations with |R| > 0.3 and P < 0.05 were considered significant. Results were visualized using the “ggplot2” package.

### 2.7 Correlation between PPAP2C expression and drug sensitivity

To investigate the relationship between PPAP2C expression and drug sensitivity, we downloaded gene expression and drug sensitivity data from the CellMiner database. Drugs were filtered to retain only FDA-approved and clinically validated agents, and the data were processed to generate numeric matrices of gene expression and drug sensitivity (IC50) values. Spearman’s rank correlation coefficients between PPAP2C expression and drug sensitivity were calculated, with |R| > 0.3 and P < 0.05 considered statistically significant. Results were visualized as scatter plots using the “ggplot2” package.

### 2.8 Gene set enrichment analysis (GSEA)

To identify biological pathways associated with PPAP2C expression, we performed GSEA using TCGA breast cancer (BRCA) data. Samples were divided into three groups based on PPAP2C expression quartiles: high-expression group (top 25%), medium-expression group (25%–75%), and low-expression group (bottom 25%). To maximize the contrast between high and low expression phenotypes, we excluded the medium-expression group. GSEA was performed using GSEA software (version 4.3.2) with the KEGG gene set (c2.cp.kegg_legacy.v2026.1.Hs.symbols.gmt) from the Molecular Signatures Database (MSigDB). Significance was determined using normalized enrichment score (NES) and false discovery rate (FDR), with FDR q < 0.05 considered statistically significant.

### 2.9 Analysis of correlation between gene expression and clinicopathological parameters

The correlation between PLPP family gene expression and clinicopathological parameters in breast cancer was conducted using the UALCAN online analysis platform (http://ualcan.path.uab.edu), which provides integrated access to The Cancer Genome Atlas (TCGA) breast cancer dataset. The analysis focused on evaluating differential expression patterns across two critical clinicopathological dimensions: pathological stages (Stage I-IV) and molecular subtypes (Luminal A, Luminal B, HER2-positive, and triple-negative breast cancer [TNBC]). For statistical comparisons among multiple groups within each dimension, one-way analysis of variance (ANOVA) was employed to detect overall significant differences in gene expression levels, with a statistical significance threshold of p < 0.05 applied to determine clinically relevant associations.

### 2.10 Survival analysis and validation

Survival analysis was systematically conducted to evaluate the prognostic significance of PLPP family members in breast cancer patients. The primary analysis was performed using the GEPIA2 platform based on The Cancer Genome Atlas (TCGA) breast cancer dataset. Kaplan-Meier curves were generated to visualize survival probability differences between patient groups stratified by high and low expression levels of PLPP family genes, with statistical significance assessed using the log-rank test. Cox proportional hazards regression analysis was employed to calculate hazard ratios (HR) and their corresponding 95% confidence intervals. To specifically validate the prognostic value of PPAP2C, independent verification was performed using both the METABRIC dataset and Gene Expression Omnibus (GEO) datasets (accession numbers GSE20685 and GSE42568). Survival analyses were performed using the survival package (version 3.2-13) in R.

### 2.11 Multivariate prognostic analysis

Main clinicopathological parameters were extracted from the TCGA database, including pathological stage, molecular subtype, treatment history, and other relevant clinical variables. The analytical approach followed a two-stage sequential design: first, univariate Cox regression analysis was performed to identify individual factors significantly associated with survival outcomes; subsequently, all statistically significant variables from the univariate analysis were incorporated into a multivariate Cox proportional hazards model to assess their independent prognostic value.

### 2.12 Tissue microarray immunohistochemistry

The breast cancer tissue microarrays (TMAs) used in this study were constructed by Shanghai Outdo Biotech Company. The study employed two distinct TMAs: the primary cancer tissue array (designated HBreD131Su08, lot number XT16-034/K16-054) and its paired normal tissue control array (HBreD077Su01). The HBreD131Su08 TMA was specifically designed for breast cancer survival studies, containing formalin-fixed, paraffin-embedded (FFPE) tissue samples from 131 breast cancer cases. Each case was represented by one tissue core (1.5 mm diameter), arranged in a standardized layout of 9 rows × 16 columns, totaling 131 points. This array included cases with complete clinicopathological data and follow-up information. Surgeries were performed between January 2005 and September 2012, with follow-up conducted until January 2016 (median follow-up 3.3–11 years). The cohort encompassed stage I–III breast cancers with diverse pathological subtypes, predominantly invasive ductal carcinoma (not otherwise specified), along with invasive lobular carcinoma, mucinous adenocarcinoma, medullary carcinoma, and other variants. The HBreD077Su01 TMA served as a matched normal control, containing 77 cases of adjacent normal breast tissue cores (1.5 mm diameter) from the same patient cohort. These samples were obtained during the same surgical procedures (2004–2008) and processed identically, providing essential baseline data for comparative analysis.

Immunohistochemical staining was performed using standardized protocols. Briefly, paraffin sections were incubated overnight at 60 °C, followed by deparaffinization in xylene and rehydration through graded ethanol series. Antigen retrieval was conducted using EDTA buffer (pH 9.0) in a pressure cooker. Primary antibody incubation employed PPAP2C polyclonal antibody (Thermo Fisher Scientific, Catalog# PA5-98075) at 1:100 dilution, incubated overnight at 4 °C. Detection was achieved using a polymer detection system with DAB chromogen development, followed by hematoxylin counterstaining, dehydration, and mounting. A semi-quantitative scoring system based on positive cell percentage was applied by two independent pathologists blinded to clinical data.

### 2.13 Cell lines and culture conditions

The human breast cancer cell lines MDA-MB-231 (Procell, Cat# CL-0150, Wuhan, Hubei, China) and MDA-MB-468 (Procell, Cat# CL-0158) were cultured under standard conditions. Both cell lines were maintained in high-glucose Dulbecco’s Modified Eagle Medium (DMEM; Gibco, Cat# C11995500BT, Waltham, MA, United States) supplemented with 10% fetal bovine serum (FBS; Gibco, Cat# 10099-141) with the addition of 1% penicillin-streptomycin (P/S; Gibco, Cat# 15140122). HEK-293T cells (ATCC, Cat# CRL-3216, Manassas, VA, United States), employed for lentiviral packaging, were cultured in the same DMEM medium containing 10% FBS with the addition of 1% P/S. All cell lines were incubated at 37 °C under a humidified atmosphere of 5% CO_2_.

### 2.14 Lentiviral plasmid construction and packaging

The coding sequence of PPAP2C was obtained from GenBank, and a FLAG tag was incorporated at its C-terminus. The resulting sequence was chemically synthesized by OBiO (Shanghai, China). The synthesized PPAP2C gene fragment was subsequently subcloned into the pLenti-EGFP-Puro plasmid using the CloneEZ method to construct the pLenti-EGFP-Puro-PPAP2C plasmid. For CRISPR/Cas9-mediated knockout, an sgRNA targeting PPAP2C (5′-AGT​TGA​CCC​GGC​TCC​AGT​CG-3′) was designed using the ChopChop online tool. This sgRNA was chemically synthesized by OBiO (Shanghai, China) and cloned into the lentiviral pLenti-Cas9 vector to generate the pLenti-Cas9-sgRNA-PPAP2C plasmid.

For lentivirus packaging, HEK-293T cells were co-transfected with 5.00 μg of either pLenti-EGFP-Puro-PPAP2C or pLenti-Cas9-sgRNA-PPAP2C, along with the packaging plasmids psPAX2 (Addgene, Cat# 12260; 3.75 μg) and pMD2.G (Addgene, Cat# 12259; 1.25 μg), using Lipofectamine 8000 transfection reagent (Beyotime, Shanghai, China). Seventy-two hours post-transfection, the lentiviral supernatants, designated as Lenti-EGFP-Puro-PPAP2C and Lenti-Cas9-sgRNA-PPAP2C, were collected, filtered through a 0.45 μm membrane, and stored at −80 °C for future use.

### 2.15 Establishment of PPAP2C-Overexpressing cell lines

MDA-MB-231 or MDA-MB-468 cells were seeded in 6-well plates at a density of 2 × 10^5^ cells per well. After 24 h, 1 mL of Lenti-EGFP-Puro-PPAP2C viral supernatant supplemented with polybrene at a final concentration of 6 μg/mL was added to the cells. The viral supernatant was replaced with fresh culture medium after 6 h of infection. Forty-eight hours post-infection, puromycin (J593, Amresco, Beijing, China) was added to the culture medium at a concentration of 2 μg/mL for selection. The selection process continued until all non-infected control cells had died. The surviving cells were washed with PBS to remove dead cells and then maintained in fresh complete medium. Following two rounds of puromycin selection, the cells were diluted to a concentration of 5 cells/mL, and 200 μL of the cell suspension was aliquoted into each well of a 96-well plate. After 2 weeks of culture, individual clones with clear, round edges were selected and sequentially expanded in 24-well plates, 6-well plates, and 90 mm culture dishes.

### 2.16 Generation of PPAP2C knockout cell lines

MDA-MB-231 or MDA-MB-468 cells were seeded in 6-well plates at a density of 2 × 10^5^ cells per well. After 24 h, the cells were infected with 1 mL of Lenti-Cas9-sgRNA-PPAP2C viral supernatant containing polybrene (Cat# H8761, Beyotime) at a final concentration of 6 μg/mL. The medium was replaced with fresh culture medium after 6 h. Puromycin selection was initiated 48 h post-infection. After two to three rounds of selection, monoclonal cells were isolated using the limited dilution method. Genomic DNA was extracted from the resulting monoclonal cell populations. The target region of the PPAP2C gene was amplified by PCR using the primers PPAP2C-F (5′-ACC​GAG​ACC​CCC​ACT​GGT​TCC​T-3′) and PPAP2C-R (5′-CTC​CAG​GGC​CTT​CTT​CAG​CTC​CCA​TTC​C-3′). The PCR products were cloned into a T-vector and subjected to Sanger sequencing (Sangon Biotech, Shanghai, China) using the M13F primer. Monoclonal cell lines carrying a 1 or 5-bp deletion in PPAP2C, which are expected to cause frameshifts and premature termination of translation, were identified through sequencing and subsequently expanded for further studies.

### 2.17 Quantitative real-time polymerase chain reaction (qPCR)

Cells were harvested, and total RNA was extracted using the Simzol reagent total RNA extraction kit (Cat# 5302100, Simgen, Hangzhou, China). First-strand cDNA was synthesized from all mRNA using an oligo-dT primer and a reverse transcription kit (Cat# M5RT04, Bioeast, Hangzhou, Zhejiang, China). The resulting cDNA was used as a template for real-time quantitative PCR (qPCR) amplification using a fluorescence quantitative PCR kit (Cat# M4QS07, Bioeast). The sequences of the specific primers used are listed in Supplementary Table S1. The primer pair designated as “PPAP2C-1” was specifically designed to span the 5-base pair deletion site in the knockout MDA-MB-231^KO^ cell line. In contrast, the primer pair designated as “PPAP2C-2” was designed to be suitable for both the MDA-MB-468^KO^ cell line (which carries a 1-base pair mutation) and the PPAP2C-overexpressing cell lines. GAPDH was used as the internal reference gene, and the relative mRNA expression levels were calculated using the ΔCt method.

### 2.18 Western blot

Cells were collected by centrifugation and lysed on ice for 30 min using 200 μL of Western blot lysis buffer (Cat# BMP 2010, Abbkin, Wuhan, China). After centrifugation at 12,000 × g for 5 min at 4 °C, the supernatant was collected and mixed with 50 μL of SDS-PAGE loading buffer (Cat# P0015, Beyotime, Shanghai, China). The samples were denatured at 95 °C–100 °C for 5 min, separated by SDS-PAGE, and transferred to a PVDF membrane (Merck Millipore, Burlington, MA, United States) at a constant current of 250 mA for 2 h. The membrane was blocked with a fast protein-free blocking buffer (Cat# PS108P, EpiZyme, Shanghai, China) at room temperature for 30 min, followed by overnight incubation at 4 °C with the following primary antibodies: PPAP2C Polyclonal Antibody (1:1,000, Cat# PA5-98075, Thermo Fisher Scientific, Waltham, MA, United States) and Beta-Actin Antibody (1:1,000, Cat# A00702, GenScript, Nanjing, China). After washing five times with TBST (20 mM Tris, 150 mM NaCl, 0.5% Tween 20, pH 7.6), the membrane was incubated for 1 h at room temperature with HRP-conjugated secondary antibodies: Goat Anti-Rabbit IgG (1:10,000, Cat# 31460, Thermo Fisher Scientific, Waltham, MA, United States) and Goat Anti-Mouse IgG (1:10,000, Cat# 31430, Thermo Fisher Scientific, Waltham, MA, United States). The membrane was then washed five times with TBST. Protein bands were visualized using an ultrasensitive ECL substrate (Cat# BMU102-CN, Abbkin, Wuhan, China) and imaged with a chemiluminescence detection system.

### 2.19 CCK-8 cell proliferation assay

The experimentally constructed cell lines (via lentiviral transduction) were seeded in 96-well plates at a density of 4,000 cells per well and cultured in DMEM supplemented with 10% FBS. Cell proliferation was monitored continuously for 1–7 days. Following the manufacturer’s instructions, the Cell Counting Kit-8 (CCK-8) solution (Cat# BMU106, Abbkin, Wuhan, China) was used to assess viability by adding the reagent to each well. After incubation at 37 °C for 1.5 h, the absorbance at 450 nm was measured using a microplate reader.

### 2.20 Colony formation assay

MDA-MB-231 cells with PPAP2C knockout (KO) or overexpression were seeded in 6-well plates at a density of 800 cells per well, while MDA-MB-468 cells with PPAP2C overexpression were seeded at 1,500 cells per well. All cells were cultured in DMEM supplemented with 10% FBS for 2–3 weeks. After visible colonies had formed, they were fixed with 4% paraformaldehyde at 4 °C for 15 min and stained with 0.1% crystal violet solution (Cat# G1063, Solarbio, Beijing, China) at room temperature for 30 min. Clonogenic ability was evaluated by counting the number of stained colonies.

### 2.21 Wound healing assay

MDA-MB-231 cells with PPAP2C knockout (KO) or overexpression were seeded in 6-well plates at 6 × 10^5^ cells per well, while MDA-MB-468 cells with PPAP2C knockout were seeded at 1 × 10^6^ cells per well. After the cells reached full confluence, a straight wound was gently created in each well using a sterile pipette tip. The wells were washed twice with PBS to remove dislodged cells, and then cultured in DMEM containing 2% FBS. Cell migration into the wound area was observed and recorded at 0, 24, and 48 h.

### 2.22 Cell migration assay

MDA-MB-231 cells with PPAP2C knockout (KO) or overexpression were seeded in the upper chamber of an 8.0 μm polycarbonate membrane insert (Cat# 3422, Corning, NY, United States) at a density of 1 × 10^4^ cells per well (24-well plate), while MDA-MB-468 cells with PPAP2C knockout or overexpression were seeded at 5 × 10^4^ cells per insert. The upper chamber contained serum-free DMEM, and the lower chamber was filled with 500 μL of DMEM supplemented with 20% FBS. After 48 h of incubation, the inserts were fixed with 4% paraformaldehyde at 4 °C for 15 min and stained with 0.1% crystal violet solution (Cat# G1063, Solarbio, Beijing, China) at room temperature for 30 min. Non-migrated cells on the upper surface were gently removed with a cotton swab. Migrated cells on the lower surface of the membrane were observed and quantified under a microscope.

### 2.23 Cell invasion assay

Transwell inserts (Cat# 3422, Corning, NY, United States) were pre-coated with 65 μL of Matrigel (working concentration: 200 μg/mL; Cat# 356234, Corning, United States) and incubated at 37 °C for 1 h to solidify. MDA-MB-231 cells with PPAP2C knockout (KO) or overexpression were seeded into the coated upper chamber at 5 × 10^4^ cells per well, while MDA-MB-468 cells with PPAP2C knockout were seeded at 1 × 10^5^ cells per insert. The upper chamber contained serum-free DMEM, and the lower chamber was filled with 500 μL of DMEM supplemented with 20% FBS. After 48 h, the inserts were fixed, stained, and processed following the same protocol as described for the migration assay. Invaded cells on the lower surface of the membrane were observed and quantified under a microscope.

### 2.24 Subcutaneous xenograft tumor model

MDA-MB-231 cells with PPAP2C knockout (KO) and their corresponding control cells in the logarithmic growth phase were harvested, washed twice with PBS, and resuspended in PBS. Cell suspensions containing 2 × 10^6^ cells in a 100 μL volume were subcutaneously injected into the fourth mammary fat pad on the left side of 7-week-old female BALB/c nude mice (Best Biotechnology, Zhuhai, China). Tumor formation was monitored beginning 12 days post-injection. Body weight and tumor dimensions (length and width) were recorded regularly. Tumor volume was calculated using the formula: V = 0.52 × L × W^2^. The experiment was terminated when the tumor volume exceeded 2000 mm^3^. All animal procedures were approved by the Institutional Animal Care and Use Committee of Youjiang Medical University for Nationalities.

### 2.25 Statistical Analysis

Statistical analyses were performed using GraphPad Prism (GraphPad Software, Boston, MA, United States) and R statistical software (version 4.1.0). Comparisons between groups were conducted using two-way ANOVA followed by *post hoc* pairwise t-tests. Non-parametric tests were applied for data that did not conform to a normal distribution. A P-value of less than 0.05 was considered statistically significant.

## 3 Results

### 3.1 Pan-cancer expression profiling of PLPP family members

**FIGURE 1:**
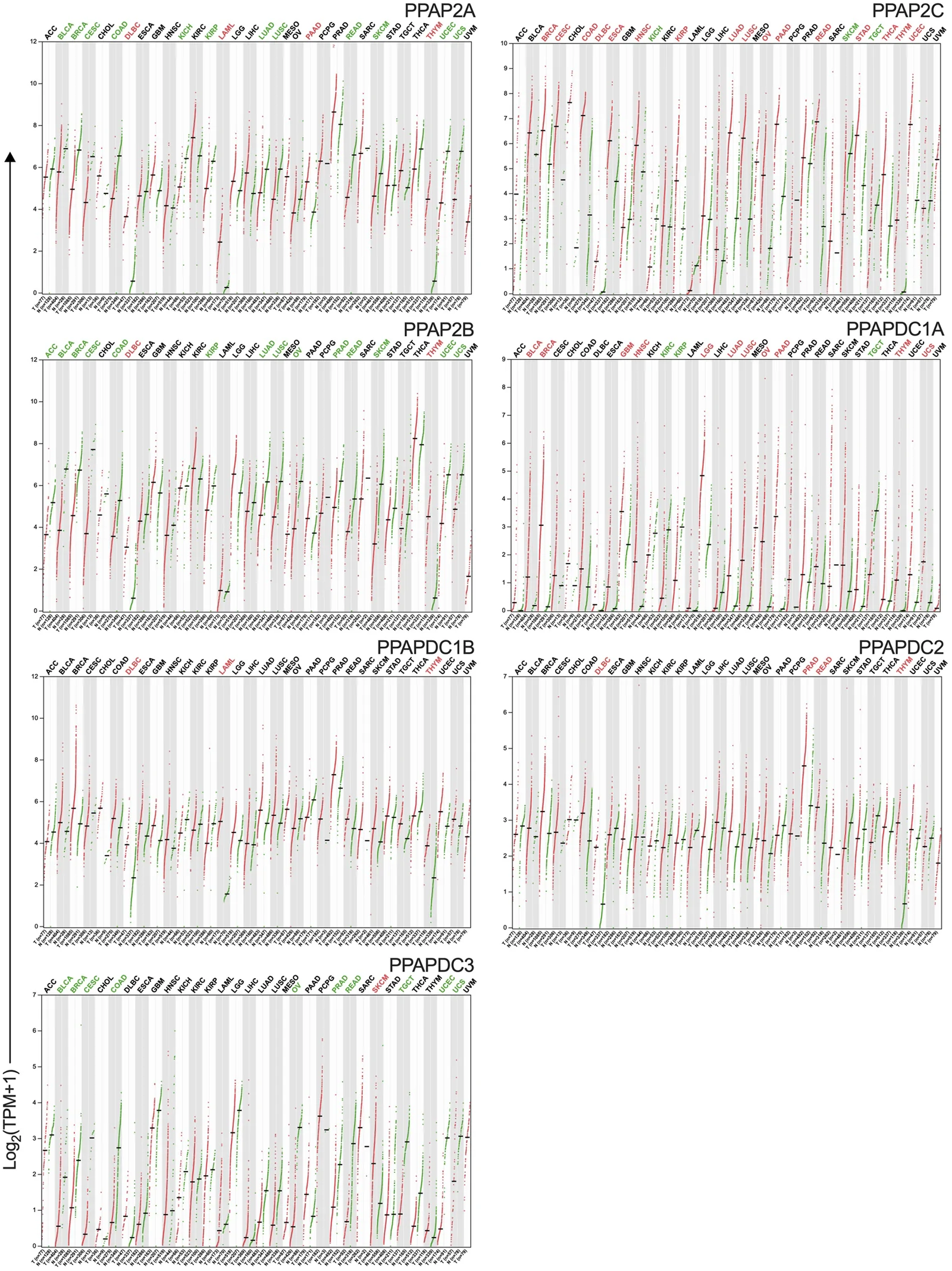
Expression of PLPP family members in pan-cancer. The mRNA expression patterns of seven PLPP family members (PPAP2A, PPAP2B, PPAP2C, PPAPDC1A, PPAPDC1B, PPAPDC2, PPAPDC3) across 33 cancer types from the TCGA database, compared to normal tissues from the GTEx database. The analysis was performed using the GEPIA2 online tool. The color coding reflects the direction and significance of expression changes: red indicates significant upregulation in tumors compared to normal tissues, green denotes significant downregulation, and black represents no statistically significant differential expression.

Using the GEPIA2 online tool, we systematically analyzed the expression patterns of PLPP family members across 33 cancer types by integrating data from TCGA and GTEx databases (Figure 1). PPAP2A was overexpressed in four cancer types (DLBC, LAML, PAAD, and THYM) but was significantly downregulated in 12 malignancies (BLCA, BRCA, CESC, COAD, KICH, KIRP, LUAD, LUSC, READ, SKCM, UCEC, and UCS). PPAP2B demonstrated elevated expression in only two cancers (DLBC and THYM), while being underexpressed in 14 tumor types (ACC, BLCA, BRCA, CESC, COAD, KIRP, LUAD, LUSC, OV, PRAD, READ, SKCM, UCEC, and UCS). PPAP2C exhibited the most widespread overexpression pattern, being upregulated in 16 cancer types (BRCA, CESC, COAD, DLBC, ESCA, HNSC, KIRP, LUAD, LUSC, OV, PAAD, READ, STAD, THCA, THYM, and UCEC), with downregulation observed in only 3 (KICH, SKCM, and TGCT). PPAPDC1A was overexpressed in 11 malignancies (BLCA, BRCA, GBM, HNSC, LGG, LUAD, LUSC, OV, PAAD, THYM, and UCS) and underexpressed in 3 (KIRC, KIRP, and TGCT). PPAPDC1B showed limited overexpression, being elevated in just three cancer types (DLBC, LAML, and THYM). PPAPDC2 was upregulated in four cancers (DLBC, PRAD, READ, and THYM). PPAPDC3 displayed the most restricted expression pattern, being overexpressed only in SKCM while downregulated in 10 other cancer types (BLCA, BRCA, CESC, COAD, OV, PRAD, READ, TGCT, UCEC, and UCS). Among the PLPP family members, PPAP2C and PPAPDC1A emerged as the most frequently upregulated members across multiple tumor types, whereas PPAP2A and PPAP2B were predominantly downregulated. Notably, PPAP2C demonstrated the most consistent upregulation in human cancers, suggesting a potentially critical role in oncogenesis.

### 3.2 Abnormal expression of PLPPs in breast cancer

**FIGURE 2:**
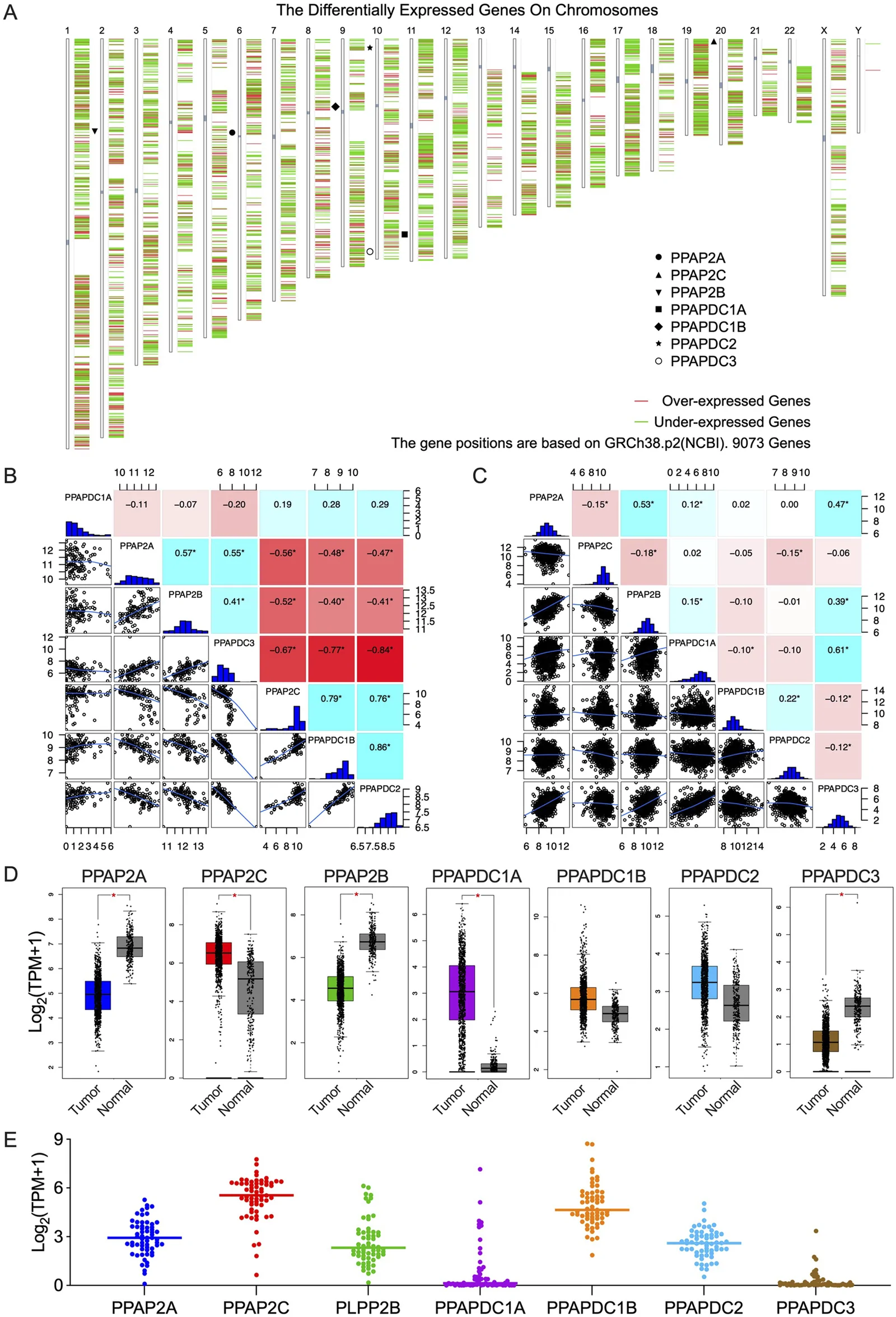
Abnormal expression of PLPPs in breast cancer. **(A)** Schematic diagram of chromosomal locations for overexpressed (red line) and underexpressed (green line) genes, including the PLPP family members. **(B)** Spearman’s correlation network of PLPP family member expression in normal breast tissue from the GTEx database. **(C)** Spearman’s correlation network of PLPP family member expression in breast cancer tissue from the TCGA database. **(D)** Box plot comparing the mRNA expression levels of PLPP family members between TCGA breast cancer tumors and GTEx normal breast tissues. **(E)** Box plot showing the mRNA expression levels of PLPP family members in breast cancer cell lines from the CCLE database. Significance is indicated by asterisks: *P < 0.05.

To investigate the expression patterns of PLPP family members in breast cancer, we compared their expression profiles between breast cancer tissues (TCGA database) and normal breast tissues (GTEx database). Chromosomal mapping revealed the genomic locations of overexpressed (red line) and underexpressed (green line) genes, including PLPP family members (Figure 2A). In normal breast tissue (GTEx), the seven PLPP genes exhibited distinct expression patterns, clustering into three groups. Group 1 consisted of a single uncorrelated gene (PPAPDC1A), while Group 2 (PPAP2A, PPAP2B, and PPAPDC3) and Group 3 (PPAP2C, PAPDC1B, and PPAPDC2) each contained three genes. Notably, genes within Groups 2 and 3 showed strong positive correlations, whereas a negative correlation was observed between the two groups (Figure 2B). However, this coordinated expression pattern was disrupted in breast cancer tissue, where no significant correlations were detected (Figure 2C). Further analysis revealed significant dysregulation of PLPP family members in breast cancer. Specifically, PPAP2C and PPAPDC1A were markedly overexpressed, whereas PPAP2A, PPAP2B, and PPAPDC3 were significantly underexpressed compared to normal tissue (Figure 2D). Consistent with these findings, PPAP2C exhibited the highest expression level among PLPP family members in breast cancer cell lines (Figure 2E). Collectively, these results demonstrate aberrant expression patterns of PLPP family members in breast cancer, suggesting their potential involvement in tumorigenesis or disease progression.

### 3.3 Association of the expression and the gene alteration of PLPPs in breast cancer

**FIGURE 3:**
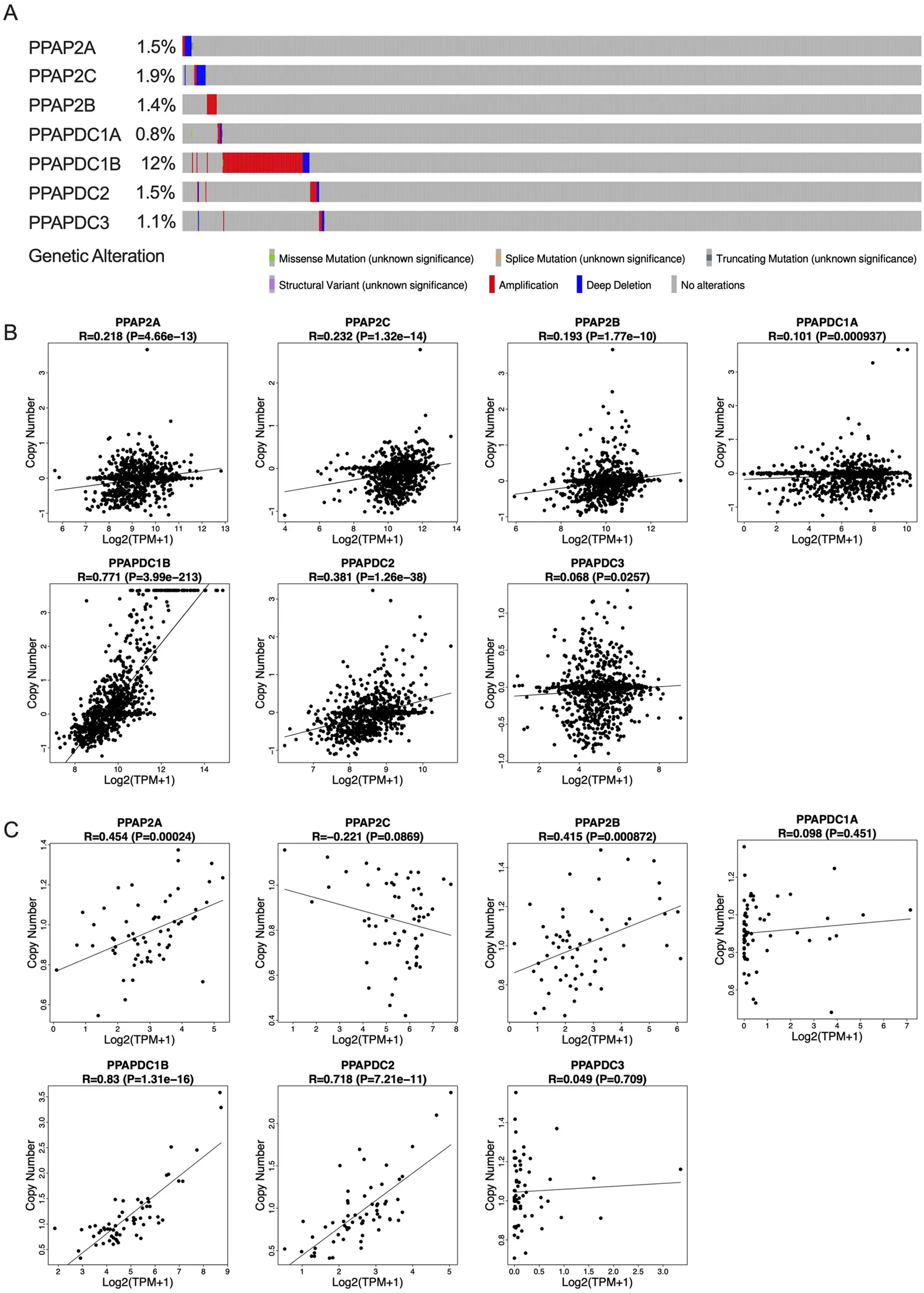
Association of the expression and the gene alteration of PLPPs in breast cancer. **(A)** OncoPrint summarizing the frequency and type of genetic alterations in PLPP family members within the TCGA breast cancer cohort, as analyzed via cBioPortal. **(B)** Scatter plots depicting the Spearman correlation between mRNA expression levels and copy number variations (CNVs) for each PLPP member in TCGA breast cancer tissues. **(C)** Scatter plots depicting the Spearman correlation between mRNA expression levels and copy number variations (CNVs) for each PLPP member in breast cancer cell lines from the CCLE database. R, Spearman’s correlation coefficients.

To investigate genetic alterations in PLPP family members, we analyzed the TCGA breast cancer dataset using cBioPortal. The analysis revealed distinct alteration patterns: PPAP2A (1.5%), PPAP2B (1.4%), PPAP2C (1.9%), PAPDC1A (0.8%), PAPDC1B (12.0%), PPAPDC2 (1.5%), and PPAPDC3 (1.1%). Strikingly, PAPDC1B showed the highest alteration frequency (12.0%). The predominant alteration types were gene amplification for PPAP2B, PAPDC1B, and PPAPDC2, while PPAP2A and PPAP2C mainly exhibited deep deletions (Figure 3A). Further analysis demonstrated that PAPDC1B and PPAPDC2 expression positively correlated with their copy numbers in both breast cancer tissues (TCGA, R > 0.3, P < 0.05; Figure 3B) and cell lines (CCLE, R > 0.7, P < 0.05; Figure 3C). Notably, although PPAP2C showed a relatively low alteration frequency (1.9%), it was significantly overexpressed in breast cancer tissues compared to normal tissues (Figure 2D), suggesting that its dysregulation may involve mechanisms other than genetic alterations.

### 3.4 Correlation between PPAP2C expression and its promoter methylation

We first compared PPAP2C promoter methylation levels between breast cancer and normal tissues using the UALCAN database. Breast tumors showed higher methylation levels (median beta = 0.604, n = 793) than normal breast tissues (median beta = 0.579, n = 97), indicating hypermethylation of the PPAP2C promoter region in breast cancer (Supplementary Figure S1A). We then asked whether this hypermethylation correlates with PPAP2C expression. Spearman’s correlation analysis using TCGA data revealed no significant relationship between promoter methylation and PPAP2C expression (R = 0.021, P = 0.56; Supplementary Figure S1B), suggesting that promoter methylation is not a major regulator of PPAP2C transcription in breast cancer.

### 3.5 Correlation between PPAP2C and breast cancer driver genes

We next examined whether PPAP2C expression correlates with the expression of 41 known breast cancer driver genes in 1,217 TCGA breast cancer samples. None of these genes met our significance threshold (|R| > 0.3, P < 0.05). Specifically, PPAP2C showed no correlation with classic drivers such as TP53, PIK3CA, and GATA3, nor with recently identified drivers including BCL11B and RREB1 (Supplementary Figure S2). These findings indicate that PPAP2C transcription is not coordinated with the breast cancer driver gene expression network.

### 3.6 Correlation between PPAP2C expression and drug sensitivity

**FIGURE 4:**
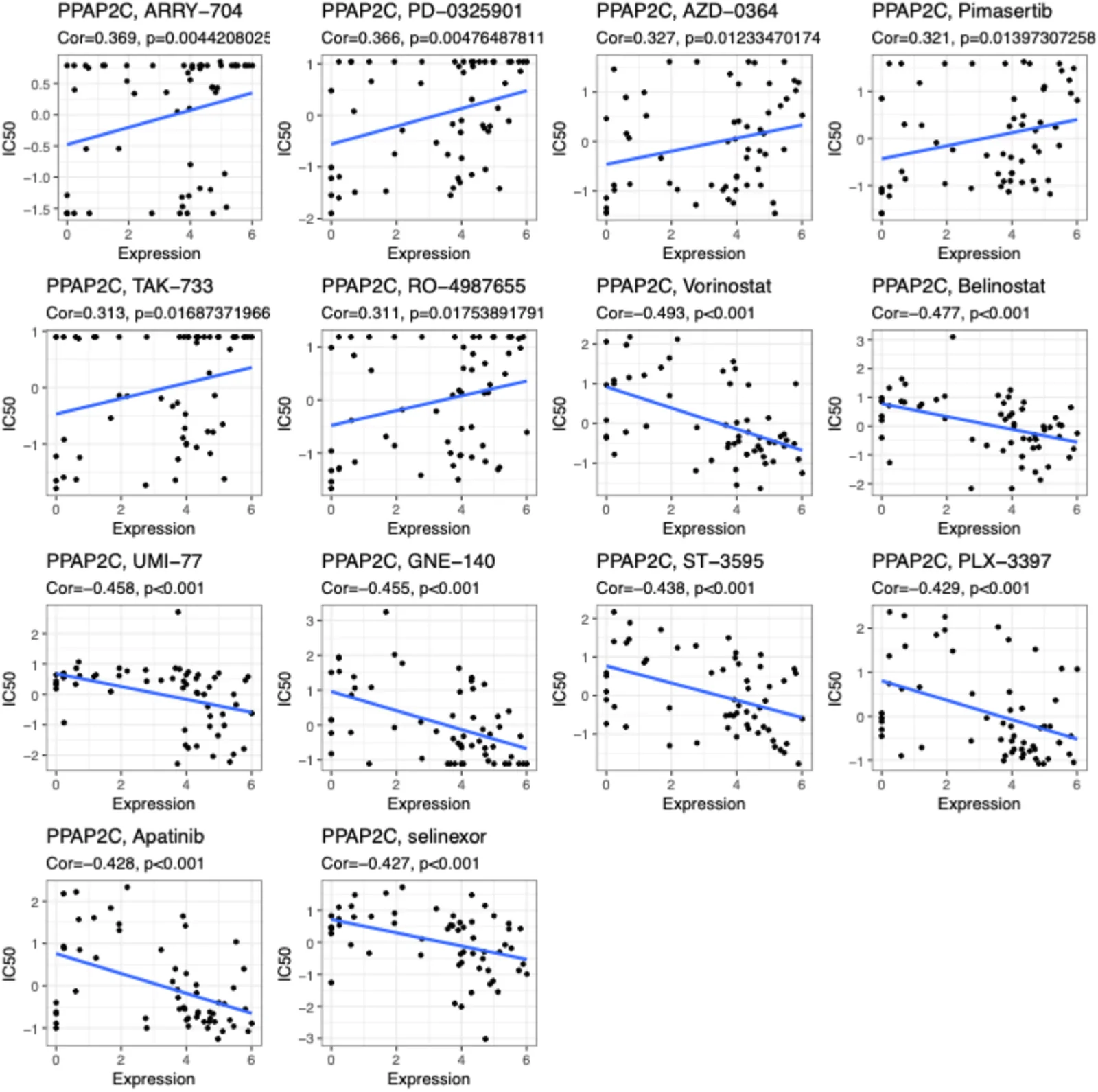
Correlation between PPAP2C expression and drug sensitivity. Scatter plots showing significant correlations between PPAP2C expression and IC50 values of 14 drugs in the CellMiner database. Spearman’s correlation coefficients (Cor) and P values are shown in each panel. |Cor| > 0.3 and P < 0.05 were considered statistically significant.

We examined the association between PPAP2C expression and drug sensitivity using the CellMiner database. A total of 14 drugs showed significant correlations with PPAP2C expression (|R| > 0.3, P < 0.05; Figure 4). PPAP2C expression was positively correlated with IC50 values of ARRY-704, PD-0325901, AZD-0364, Pimasertib, TAK-733, and RO-4987655, indicating that higher PPAP2C expression was associated with decreased sensitivity to these MEK/ERK pathway inhibitors. In contrast, negative correlations were observed between PPAP2C expression and IC50 values of Vorinostat, Belinostat, UMI-77, GNE-140, ST-3595, PLX-3397, Apatinib, and Selinexor, suggesting that higher PPAP2C expression was linked to increased sensitivity to these drugs. Among them, Vorinostat (R = −0.493, P < 0.001) showed the strongest negative correlations, whereas ARRY-704 (R = 0.369, P = 0.004) exhibited the strongest positive correlations. These results suggest that PPAP2C expression may influence tumor cell responses to a range of therapeutic agents.

### 3.7 Gene set enrichment analysis of PPAP2C expression

After excluding the medium-expression group, 270 tumor samples remained in each of the high- and low-expression groups. GSEA revealed that 40 KEGG pathways were significantly enriched in the PPAP2C high-expression group (FDR <0.05), whereas no pathways reached significance in the low-expression group (Supplementary Figure S3). The enriched pathways in the high-expression group could be broadly categorized into five functional themes. First, pathways related to DNA replication and repair were significantly enriched, including base excision repair (NES = 2.096, FDR = 0.002), homologous recombination (NES = 1.997, FDR = 0.006), mismatch repair (NES = 1.836, FDR = 0.027), nucleotide excision repair (NES = 1.774, FDR = 0.036), and DNA replication (NES = 1.909, FDR = 0.015). Second, pathways involved in RNA processing and protein turnover were overrepresented, including spliceosome (NES = 2.216, FDR <0.001), RNA polymerase (NES = 2.083, FDR = 0.002), RNA degradation (NES = 1.923, FDR = 0.013), ribosome (NES = 1.877, FDR = 0.021), and proteasome (NES = 2.002, FDR = 0.006). Third, pathways related to cell cycle progression were enriched, including cell cycle (NES = 2.081, FDR = 0.001) and oocyte meiosis (NES = 1.829, FDR = 0.027). Fourth, multiple metabolic pathways were significantly enriched, including pyrimidine metabolism (NES = 2.092, FDR = 0.002), purine metabolism (NES = 2.034, FDR = 0.004), pentose phosphate pathway (NES = 2.108, FDR = 0.002), oxidative phosphorylation (NES = 2.110, FDR = 0.002), selenoamino acid metabolism (NES = 2.123, FDR = 0.002), glutathione metabolism (NES = 1.942, FDR = 0.013), and citrate cycle (NES = 1.800, FDR = 0.032). Finally, several cancer-related signaling pathways were enriched, including the ErbB signaling pathway (NES = 1.741, FDR = 0.044), Notch signaling pathway (NES = 1.847, FDR = 0.025), and bladder cancer (NES = 1.824, FDR = 0.027). Collectively, these findings indicate that high PPAP2C expression is associated with enhanced DNA replication and repair, RNA processing, cell proliferation, metabolic reprogramming, and oncogenic signaling, suggesting a potential role for PPAP2C in promoting tumor cell proliferation and coping with replication stress.

### 3.8 Association of the mRNA expression of PLPPs with clinicopathological features in breast cancer

**FIGURE 5:**
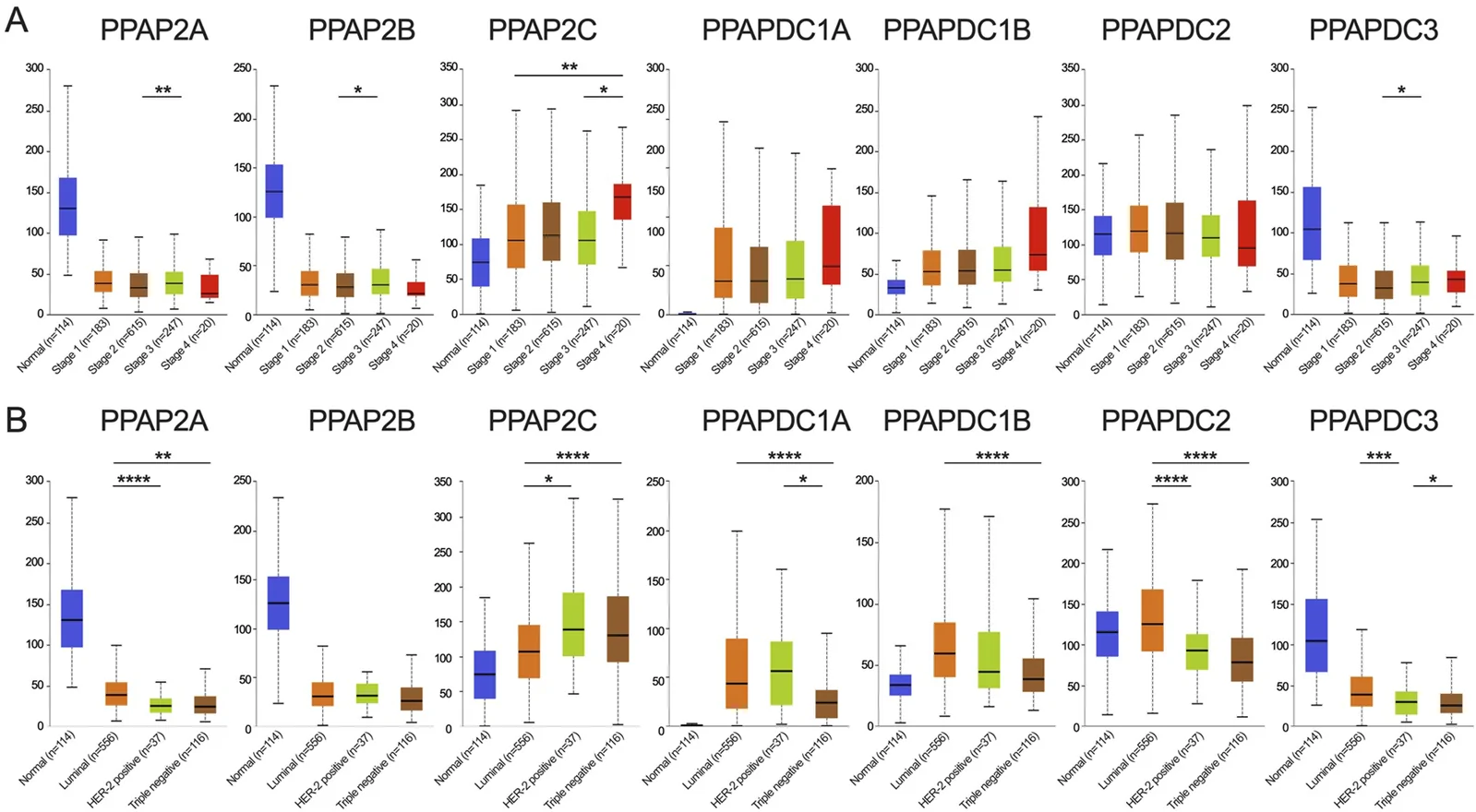
Association of the mRNA expression of PLPPs and cli-pathologic features. Analysis performed using the TCGA breast cancer dataset via the UALCAN platform. **(A)** Box plot comparing the mRNA expression levels of PLPPs across different pathological stages (I–IV). **(B)** Box plot comparing the mRNA expression levels of PLPPs across different molecular subtypes (Luminal, HER2-positive, Triple-negative breast cancer). Significance is indicated by asterisks: *P < 0.05, **P < 0.01, ***P < 0.001, ****P < 0.0001.

Utilizing the TCGA breast cancer transcriptomic and clinical dataset, we investigated the differential expression patterns of PLPPs across disease stages and molecular subtypes. Our analysis revealed that PPAP2A, PPAP2B, and PPAPDC3 expression levels were significantly elevated in stage III patients compared to stage II cases. Notably, PPAP2C demonstrated substantially higher expression in stage IV tumors relative to both stage I and III specimens (Figure 5A). At the molecular subtype level, PPAP2C exhibited significantly increased expression in HER2-enriched and TNBC compared to Luminal subtypes (Luminal A/B). In contrast, other family members displayed inverse expression trends: PPAP2A, PPAPDC1A, PPAPDC1B, and PPAPDC2 showed significantly higher expression in Luminal subtypes versus TNBC, while PPAPDC2 expression was also markedly elevated in Luminal subtypes compared to HER2-enriched tumors. Additionally, PPAPDC1A and PPAPDC3 demonstrated higher expression levels in HER2-enriched tumors than in TNBC cases (Figure 5B). The distinct expression profile of PPAP2C, particularly its upregulation in advanced-stage disease and aggressive molecular subtypes, positions it as a compelling PPAP2 family candidate for both functional characterization and biomarker development.

### 3.9 Prognostic significance of PLPPs in breast cancer

**FIGURE 6:**
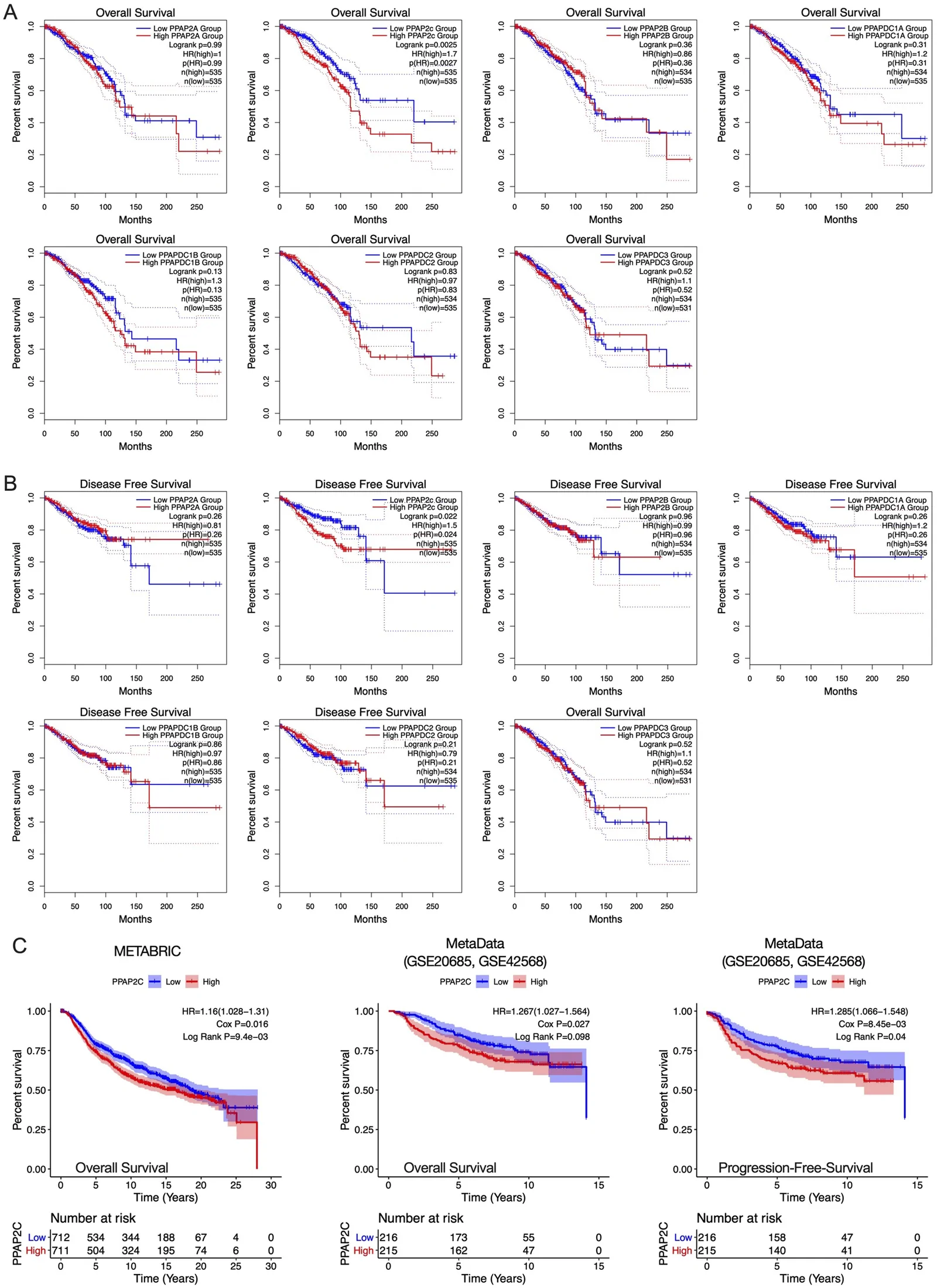
Prognostic values of the PLPPs in breast cancer patients. **(A)** Kaplan-Meier curves for overall survival (OS) of breast cancer patients in the TCGA dataset, stratified by high and low expression of each PLPP family member. **(B)** Kaplan-Meier curves for disease-free survival (DFS) of breast cancer patients in the TCGA dataset, stratified by high and low expression of each PLPP family member. **(C)** Kaplan-Meier curves validating the prognostic value of PPAP2C expression for overall survival (OS) and progression-free survival (PFS) in independent validation cohorts (METABRIC and GEO datasets).

Through comprehensive analysis of the TCGA dataset using GEPIA2, we evaluated the prognostic potential of PLPP family members in breast cancer. Strikingly, among the seven PLPP genes examined, only PPAP2C emerged as a significant prognostic marker, with elevated expression correlating with poorer overall survival (OS; HR = 1.7, P = 0.0027; Figure 6A) and reduced disease-free survival (DFS; HR = 1.5, P = 0.024; Figure 6B). This association was consistently validated across multiple independent cohorts: in the METABRIC dataset, high PPAP2C expression predicted worse OS (HR = 1.16, P = 0.016), while analysis of combined GEO datasets (GSE20685 and GSE42568) revealed significant associations with both diminished OS (HR = 1.267, P = 0.027) and progression-free survival (PFS; HR = 1.285, P = 0.008; Figure 6C). These robust, reproducible findings across diverse patient populations establish PPAP2C as a unique prognostic indicator among PLPP family members, highlighting its potential clinical utility for risk stratification in breast cancer management. The consistent association of PPAP2C overexpression with adverse outcomes suggests its possible involvement in aggressive tumor biology, warranting further mechanistic investigation.

### 3.10 Tissue microarray validates PPAP2C as a prognostic biomarker in breast cancer

**FIGURE 7:**
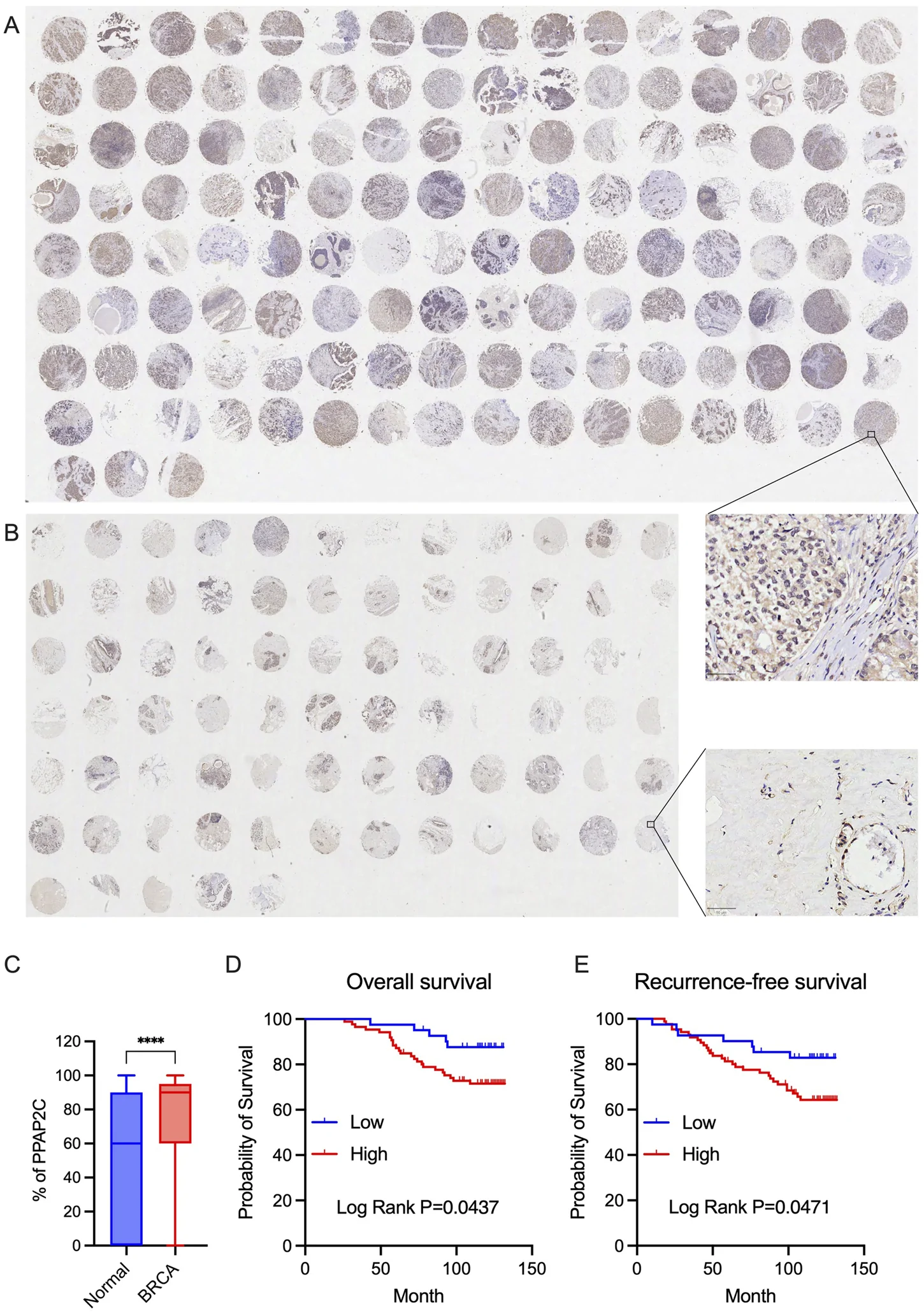
Tissue microarray analysis validates PPAP2C overexpression and its prognostic significance in breast cancer. **(A)** Representative immunohistochemistry (IHC) staining image showing PPAP2C protein expression in breast cancer tissue. **(B)** Representative IHC staining image showing PPAP2C protein expression in adjacent normal breast tissue. **(C)** Bar graph comparing the positivity rate of PPAP2C protein expression between breast cancer tissues and adjacent normal tissues. **(D)** Kaplan-Meier curve for overall survival (OS) of breast cancer patients stratified by high and low PPAP2C protein expression. **(E)** Kaplan-Meier curve for recurrence-free survival (RFS) of breast cancer patients stratified by high and low PPAP2C protein expression. Significance is indicated by asterisks: ****P < 0.0001.

Building upon the transcriptomic findings, we conducted protein-level validation using breast cancer tissue microarrays (Shanghai Outdo Biotech, Shanghai, China). Immunohistochemical staining revealed pronounced cytoplasmic PPAP2C expression in tumor cells, with significantly higher detection rates in malignant tissues (94.7%, 124/131) compared to adjacent normal tissue (59.7%, 46/77; P < 0.0001; Figures 7A–C). Importantly, Kaplan-Meier survival analysis demonstrated that patients with high PPAP2C expression had significantly worse overall survival (OS) and recurrence-free survival (RFS) than those with low expression (Log-rank P < 0.05 for both; Figures 7D,E). These protein-level observations not only validate our previous transcriptomic prognostic analyses but also establish PPAP2C as a consistent biomarker across different molecular platforms, highlighting its clinical relevance in breast cancer prognosis.

### 3.11 PPAP2C serves as an independent prognostic factor for overall survival in breast cancer patients

**FIGURE 8:**
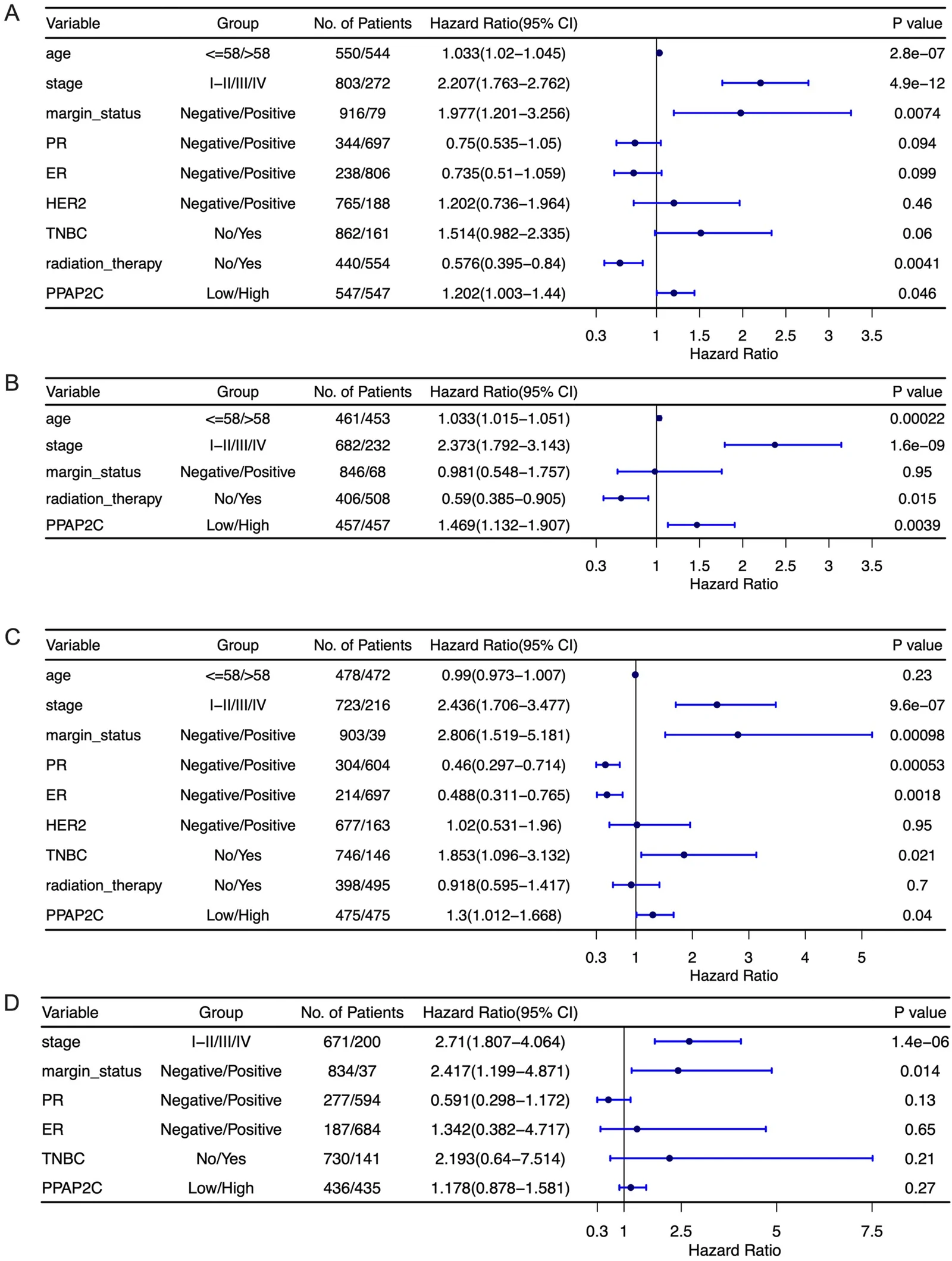
Prognostic value of PPAP2C in breast cancer patients by univariate and multivariate Cox regression analyses. Forest plots from Cox regression analyses performed on the TCGA breast cancer cohort. **(A)** Univariate analysis of clinicopathological factors and PPAP2C expression for overall survival (OS). **(B)** Multivariate analysis of clinicopathological factors and PPAP2C expression for OS. **(C)** Univariate analysis of clinicopathological factors and PPAP2C expression for progression-free survival (PFS). **(D)** Multivariate analysis of clinicopathological factors and PPAP2C expression for PFS.

To assess the prognostic significance of PPAP2C, we performed systematic survival analyses in breast cancer patients. Univariate Cox regression revealed that pathological stage (*P* < 0.0001), margin status (*P* < 0.01), radiation therapy (*P* < 0.01), and high PPAP2C expression (*P* < 0.05) were significantly associated with worse overall survival (OS) (Figure 8A). Importantly, in multivariate analysis adjusting for these established risk factors, PPAP2C expression retained independent prognostic value for OS (HR = 1.469, 95% CI 1.132–1.907, *P* = 0.0039) (Figure 8B). For progression-free survival (PFS), univariate analysis identified pathological stage (P < 0.0001), margin status (P < 0.001), triple-negative breast cancer (TNBC) subtype (P < 0.05), and PPAP2C expression (P < 0.05) as adverse prognostic factors, while ER-positive (P < 0.001) and PR-positive (P < 0.001) status were protective (Figure 8C). However, PPAP2C did not retain independent prognostic significance for PFS in the multivariate model (Figure 8D). Together, these results demonstrate that PPAP2C is an independent predictor of poor overall survival in breast cancer, highlighting its potential clinical utility as a prognostic biomarker.

### 3.12 PPAP2C enhances proliferative capacity and colony formation of TNBC cells

**FIGURE 9:**
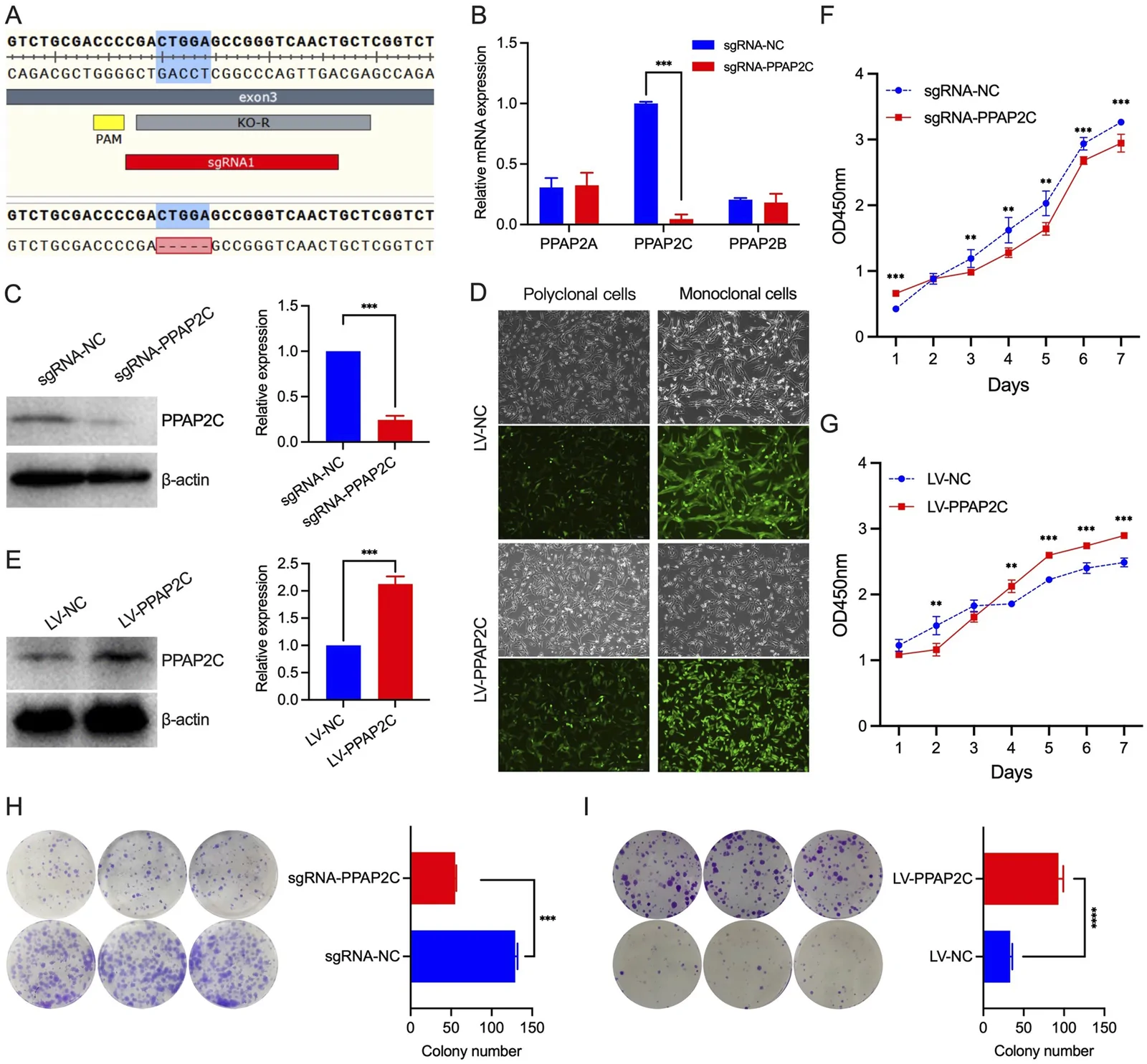
PPAP2C regulates proliferative capacity and colony formation in MDA-MB-231 cells. **(A)** Schematic illustration of the CRISPR-Cas9-mediated knockout strategy for PPAP2C, accompanied by sequencing chromatograms confirming frameshift mutations in monoclonal MDA-MB-231 cell lines. **(B)** Quantitative PCR (qPCR) analysis employing gene-specific primers to validate PPAP2C transcript knockdown and examine expression levels of additional PLPP family members. **(C)** Western blot analysis comparing PPAP2C protein expression between knockout and control cell lines. **(D)** CCK-8 proliferation assay monitoring growth dynamics of PPAP2C-knockout and control cells. **(E)** Colony formation assay evaluating the clonogenic potential of PPAP2C-knockout cells plated at low density. **(F)** Representative bright-field and fluorescence (EGFP) images of PPAP2C-overexpressing MDA-MB-231 cell lines established through lentiviral vector transduction and monoclonal selection. **(G)** Western blot analysis verifying PPAP2C protein overexpression in lentivirus-transduced and control cells. **(H)** CCK-8 proliferation assay monitoring growth dynamics of PPAP2C-overexpressing and control cells. **(I)** Colony formation assay comparing the clonogenicity between PPAP2C-overexpressing and control cells. Significance is indicated by asterisks: *P < 0.05, **P < 0.01, ***P < 0.001, ****P < 0.0001.

To elucidate the functional role of PPAP2C in TNBC, we performed CRISPR-Cas9 genome editing to generate PPAP2C frameshift-mutated monoclonal cell lines. In MDA-MB-231 cells, a 5-bp deletion was created, while MDA-MB-468 cells received a 1-bp insertion, both of which produced premature termination (knockout) and were verified by DNA sequencing (Figure 9A and Supplementary Figure S4A). As controls, the mRNA levels of PPAP2A and PPAP2B showed no significant differences between PPAP2C-knockout MDA-MB-231 cells and control cells stably expressing non-targeting sgRNAs (Figure 9B). However, quantitative PCR (qPCR) analysis using primers spanning the edited regions demonstrated significantly reduced PPAP2C transcript levels in PPAP2C-knockout cells compared to control cells (Figure 9B and Supplementary Figure S4B). Western blot analysis further confirmed dramatically reduced levels of full-length PPAP2C protein in these PPAP2C knockout cells (Figure 9C). Functional assessment of these PPAP2C-knockout cells revealed substantial impairments in key oncogenic properties. Both cellular proliferation (as measured by CCK-8 assay; Figure 9D and Supplementary Figure S4C) and colony formation capacity (Figure 9E) were markedly diminished in PPAP2C-knockout cells. In parallel, we generated stable PPAP2C-overexpressing cell lines in both MDA-MB-231 and MDA-MB-468 backgrounds through lentiviral transduction followed by monoclonal selection (Figure 9F and Supplementary Figure S4D). Successful PPAP2C overexpression was confirmed at both transcriptional (Supplementary Figure S4E) and translational levels (Figure 9G) by qPCR and Western blot, respectively. Notably, PPAP2C overexpression produced reciprocal phenotypic effects, significantly enhancing cellular proliferation (Figure 9H and Supplementary Figure S4F) and colony formation efficiency (Figure 9I and Supplementary Figure S4G) compared to controls.

### 3.13 PPAP2C drives TNBC cell migration and invasion

**FIGURE 10:**
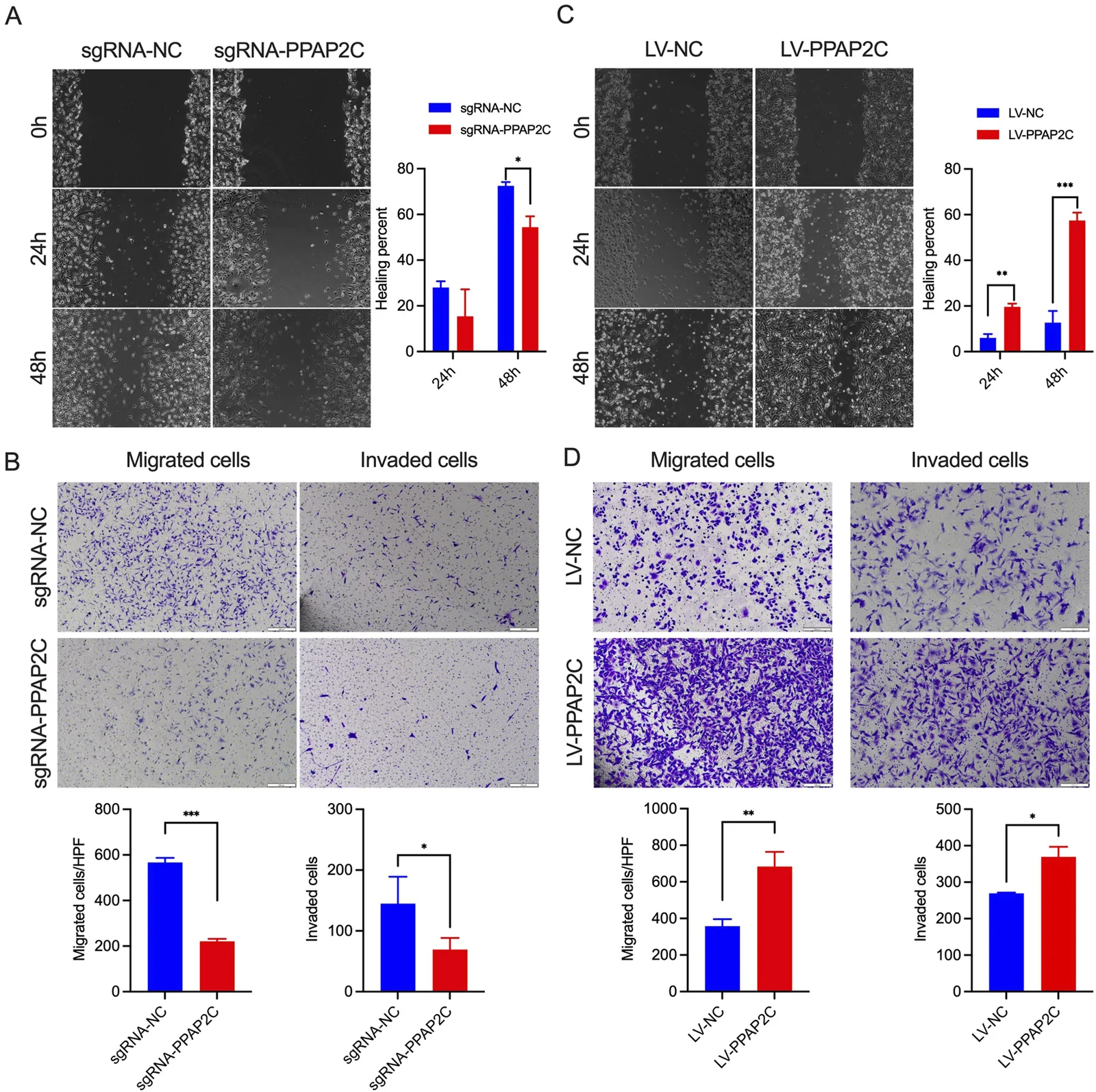
PPAP2C promotes migration and invasion of MDA-MB-231 cells. **(A)** Wound healing assay comparing the migration capabilities of PPAP2C-knockout and control MDA-MB-231 cells. **(B)** Transwell invasion assay evaluating the migration and invasion potentials of PPAP2C-knockout and control MDA-MB-231 cells. **(C)** Wound healing assay comparing the migration capabilities of PPAP2C-overexpressing and control MDA-MB-231 cells. **(D)** Transwell invasion assay evaluating the migration and invasion potentials of PPAP2C-overexpressing and control MDA-MB-231 cells. Significance is indicated by asterisks: *P < 0.05, **P < 0.01, ***P < 0.001.

To assess the role of PPAP2C in TNBC metastasis, we performed wound healing and Trans-well invasion assays. Genetic ablation of PPAP2C using CRISPR-Cas9 markedly suppressed the migration and invasion of MDA-MB-231 and MDA-MB-468 cells (Figures 10A,B and Supplementary Figure S5A). Conversely, lentivirus-mediated PPAP2C overexpression substantially promoted both migratory and invasive capacities in TNBC cells (Figures 10C,D and Supplementary Figure S5C). These results unequivocally establish PPAP2C as a critical regulator of TNBC metastatic progression.

### 3.14 Knockout of PPAP2C induces tumor regression in TNBC xenograft models

**FIGURE 11:**
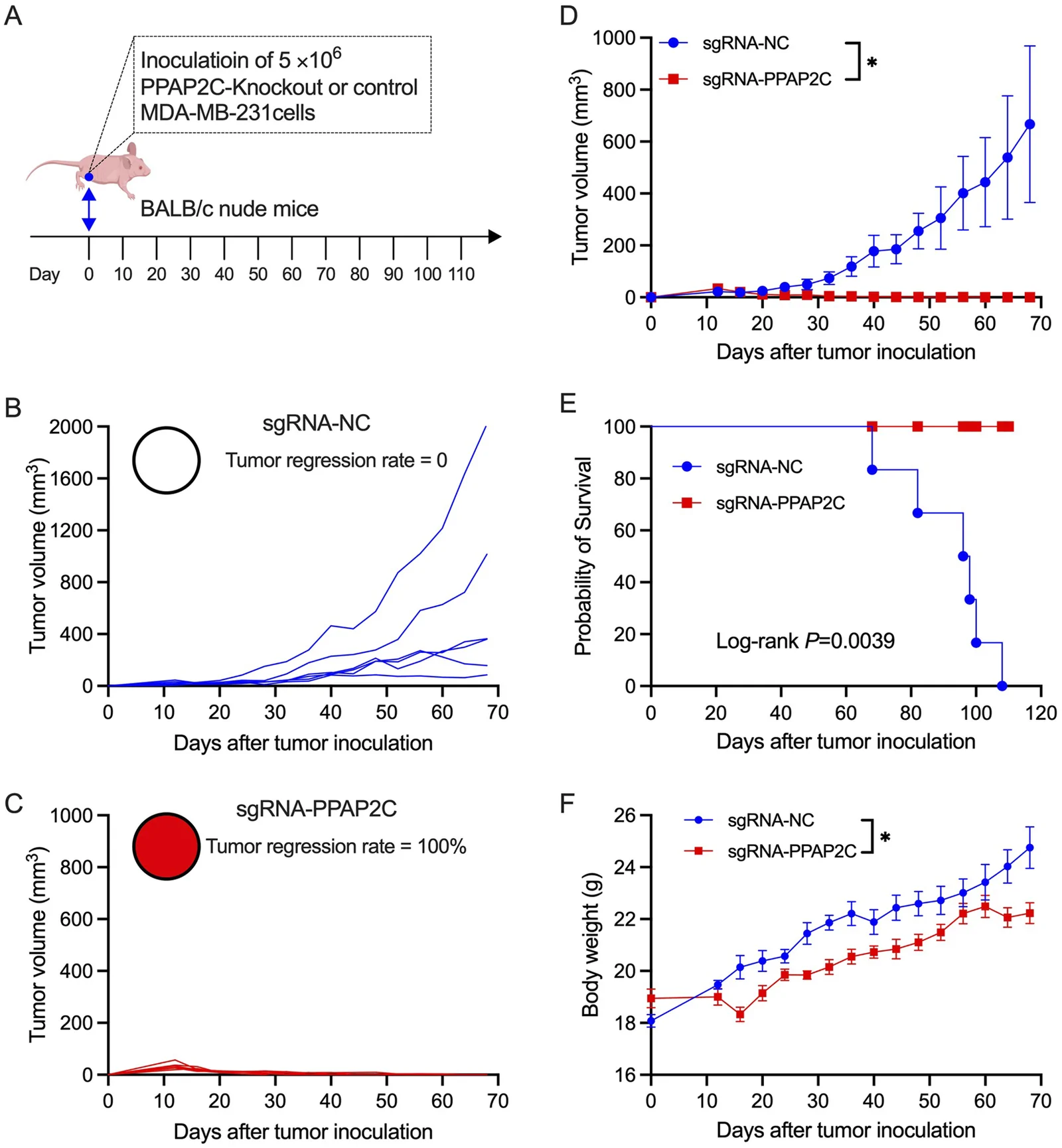
Knockout of PPAP2C induces tumor regression in MDA-MB-231xenograft models. **(A)** Schematic diagram of the experimental design, illustrating the orthotopic injection of PPAP2C-knockout or control MDA-MB-231 cells into the mammary fat pads of immunocompromised nude mice. **(B,C)** Individual tumor growth curves for representative mice from the control and PPAP2C-knockout groups, respectively. **(D)** Analysis of mean tumor volume within each experimental group, presented as the average ±standard deviation (SD) at each measured time point. **(E)** Kaplan-Meier survival curve comparing the percentage of survival between the control and PPAP2C-knockout groups. **(F)** Body weight measurements of mice. Significance is indicated by asterisks: *P < 0.05.

To investigate the functional role of PPAP2C in TNBC progression *in vivo*, we established an orthotopic xenograft model by implanting CRISPR-Cas9-mediated PPAP2C-knockout and control MDA-MB-231 cells into the mammary fat pads of BALB/c nude mice (Figure 11A). All mice implanted with control cells developed tumors without spontaneous regression (0/6, 0%) (Figure 11B). Strikingly, after a brief period of tumor formation, all mice receiving PPAP2C-knockout cells exhibited tumor regression and eventual disappearance (7/7, 100%) (Figure 11C). Comparative analysis revealed statistically significant differences (P < 0.05) between the two groups in terms of tumor size, survival time, and body weight changes (Figures 11D–F). These findings conclusively demonstrate that PPAP2C loss-of-function completely abolishes the tumorigenic capacity of MDA-MB-231 cells, identifying PPAP2C as both a key driver of TNBC progression and a potential therapeutic target.

## 4 Discussion

This study represents the first comprehensive analysis to establish the phospholipid phosphatase family member PPAP2C (also known as PLPP2) as a high-value prognostic biomarker and a potent oncogene in breast cancer. Our systematic pan-cancer analysis highlighted a striking and specific upregulation of PPAP2C in breast cancer tissues, a finding that stands in contrast to the more varied expression patterns of other PLPP family members (Benesch et al., 2023; Bernard-Pierrot et al., 2008). The clinical significance of this overexpression is profound; we demonstrated a robust association between high PPAP2C levels and adverse clinicopathological features, including advanced tumor stage and classification into HER2-positive and Triple-Negative Breast Cancer (TNBC) subtypes. Crucially, this correlation translated into significantly poorer patient outcomes, with high PPAP2C expression independently predicting diminished overall and disease-free survival across multiple large-scale cohorts (TCGA, METABRIC, GEO) and our own tissue microarray validation. The functional necessity of PPAP2C for tumor progression was unequivocally demonstrated by our *in vitro* experiments, where its knockout suppressed malignant phenotypes, and was dramatically underscored by the complete regression of established TNBC xenografts *in vivo* upon PPAP2C knockout. Collectively, these findings move beyond simple correlation to establish a causal role for PPAP2C in breast cancer pathogenesis, positioning it as a pivotal regulator of tumor growth and metastasis and, consequently, an exceptionally promising therapeutic target.

The expression dichotomy within the phospholipid phosphatase family is particularly noteworthy, where PPAP2C acts as a driver of oncogenesis, PPAP2A and PPAP2B appear to function as metabolic guardians, with their loss contributing to cancer progression through disrupted lipid signaling homeostasis (Benesch et al., 2023; Jose and Kienesberger, 2021). This inverse relationship is further supported by our co-expression network analysis, which identified distinct gene groups with opposing expression patterns: Group 2 (PPAP2A, PPAP2B, and PPAPDC3) and Group 3 (PPAP2C, PPAPDC1B, and PPAPDC2) demonstrated strong positive correlations within each group but negative correlations between groups. Importantly, this coordinated expression pattern is disrupted in breast cancer tissue, where no significant correlations were detected, suggesting a fundamental rewiring of lipid signaling homeostasis in malignancy. The mechanistic basis underlying PPAP2C overexpression in breast cancer remains incompletely understood. Our integrative analysis systematically evaluated several potential upstream regulatory mechanisms. First, although PPAP2C promoter methylation levels were elevated in breast tumors compared with normal tissues, no correlation was observed between methylation status and PPAP2C mRNA expression, suggesting that promoter hypermethylation is unlikely to be the primary driver of PPAP2C transcriptional activation. Second, PPAP2C expression showed no significant correlation with the expression of 41 known breast cancer driver genes, nor was it associated with gene amplification or deletion, as the frequency of PPAP2C genetic alterations was low (1.9%) in the TCGA cohort. Collectively, these findings indicate that PPAP2C overexpression in breast cancer is not attributable to promoter methylation, coordination with canonical driver genes, or copy number variation. The mechanisms governing PPAP2C upregulation therefore remain to be elucidated.

The oncogenic function of PPAP2C extends beyond breast cancer, demonstrating a conserved role in driving tumor progression across malignancies. In lung adenocarcinoma, PPAP2C promotes lipid raft formation and cell cycle progression, with elevated expression correlating with poor prognosis (Wang et al., 2023). A recent study demonstrated the role of PPAP2C in promoting breast cancer growth through regulation of the c-Myc pathway by investigating its overexpression in non-malignant cells and employing a knockout strategy in malignant cells (Tang et al., 2022). In our current study, we took a more targeted approach by performing both overexpression and complete knockout of PPAP2C in the same triple-negative breast cancer (TNBC) cell line. This bidirectional genetic validation within an isogenic background effectively eliminates interference from cell line-specific variations, providing more rigorous evidence for the oncogenic function of PPAP2C. Notably, complete knockout of PPAP2C not only significantly suppressed tumor cell proliferation, migration, invasion, and colony formation but also led to the complete regression of established xenograft tumors. This more pronounced phenotypic outcome suggests that PPAP2C may possess deeper therapeutic potential than previously recognized. The observed difference in efficacy may be closely related to the choice of animal models: while the previous study utilized highly immunocompromised NSG mice (Tang et al., 2022), our experiments were conducted in nude mice with residual immune function. The observation of tumor regression in a model retaining innate immunity implies that the anti-tumor effects of PPAP2C ablation may extend beyond cell-autonomous mechanisms to potentially involve activation of the host immune system. This mechanistic insight opens new avenues for exploring combination strategies targeting PPAP2C alongside immunotherapeutic approaches.

The central mechanism underlying PPAP2C’s pro-tumorigenic activity likely resides in its enzymatic function as a lipid phosphatase, which regulates the bioavailability of potent signaling lipids in the tumor microenvironment (Benesch et al., 2023). PPAP2C is known to dephosphorylate extracellular bioactive lipids such as phosphatidic acid (PA) and lysophosphatidic acid (LPA), converting them into diacylglycerol (DAG) and monoacylglycerol, respectively (Brindley and Waggoner, 1998). Both PA and LPA are powerful mitogens and chemoattractants that signal through G-protein coupled receptors (GPCRs) to drive cancer cell proliferation, survival, migration, and invasion (Mills and Moolenaar, 2003). While it seems paradoxical that the overexpression of an enzyme that degrades pro-tumorigenic lipids would promote cancer, the biological context is critical. PPAP2C is an ecto-enzyme, primarily regulating the extracellular lipid pool (Jose and Kienesberger, 2021). Its overexpression could create steep concentration gradients of LPA, enhancing chemosensory responses and directed cell migration. Alternatively, the product of PA dephosphorylation, DAG, is itself a critical second messenger that can activate protein kinase C (PKC) isoforms and RasGRP proteins, which are central hubs in oncogenic signaling (Cooke and Kazanietz, 2022). In this study, GSEA revealed that multiple KEGG pathways were enriched in breast cancer samples with high PPAP2C expression. These pathways were primarily associated with DNA replication and repair (including base excision repair, homologous recombination, mismatch repair, nucleotide excision repair, and DNA replication), RNA processing and protein turnover (spliceosome, RNA polymerase, RNA degradation, ribosome, and proteasome), cell cycle progression, metabolic pathways (oxidative phosphorylation, pentose phosphate pathway, pyrimidine metabolism, purine metabolism, glutathione metabolism, and citrate cycle), as well as ErbB and Notch signaling pathways. This enrichment profile suggests an association between high PPAP2C expression and enhanced genomic maintenance, biosynthetic metabolism, and cell proliferation signals. Furthermore, the proliferative and metabolic features reflected by the above enriched pathways are generally consistent with the observed phenotypes of PPAP2C-promoted proliferation, migration, and invasion in TNBC cells *in vitro*, as well as tumor growth *in vivo*, providing additional support for a potential oncogenic role of PPAP2C in breast cancer. Despite the comprehensive characterization of PPAP2C-associated pathways by GSEA, the downstream signaling cascades through which PPAP2C exerts its oncogenic functions were not systematically interrogated in the present study. Further investigations are warranted to identify the critical downstream effectors and signaling nodes that mediate PPAP2C-driven phenotypes in breast cancer.

The clinical implications of this study are threefold. First, as a robust and independent prognostic biomarker, PPAP2C expression status could be integrated into existing risk stratification models for breast cancer patients. Its particularly high expression in TNBC, a subtype notoriously lacking targeted therapies (Xu et al., 2025), suggests it could help identify patients at the highest risk of recurrence who may benefit from more aggressive adjuvant therapies or inclusion in clinical trials for novel agents. Second, our drug sensitivity analysis revealed that PPAP2C expression correlates with the efficacy of several clinically available agents. Specifically, PPAP2C-high tumors showed positive correlations with IC50 values of MEK inhibitors (PD-0325901, Pimasertib, and TAK-733) and the ERK inhibitor AZD-0364, suggesting potential intrinsic resistance to MEK/ERK pathway blockade. Conversely, negative correlations were observed with histone deacetylase inhibitors (vorinostat and belinostat) and selinexor (an inhibitor of the nuclear export receptor exportin 1), indicating that PPAP2C-high tumors may be more sensitive to these agents. These findings suggest that PPAP2C expression status may serve as a predictive biomarker for therapy selection, complementing its prognostic utility. Third, the xenograft regression upon PPAP2C knockout suggests a therapeutic angle worth exploring. Although its extracellular domain is theoretically amenable to biologic targeting (Flanagan et al., 2009), this notion remains preliminary and awaits rigorous validation. Our study offers a starting point, and further investigations are needed to determine whether PPAP2C is truly druggable.

## 5 Conclusions

This study systematically elucidates the dual value of PPAP2C in breast cancer prognosis and treatment through integrated multi-omics analysis and functional experiments. On one hand, PPAP2C overexpression is significantly associated with advanced clinical stage, aggressive molecular subtypes (HER2-positive and TNBC), and poor prognosis, independent of conventional pathological factors such as tumor stage and receptor status, establishing its clinical utility as a novel prognostic biomarker. On the other hand, PPAP2C drives tumor progression by enhancing proliferation, clonogenicity, migration, and invasion capabilities, while CRISPR-Cas9 knockout completely reverses tumorigenicity *in vivo*, confirming its feasibility as a therapeutic target. Future studies should further explore its downstream effectors and interactions with other oncogenic pathways, such as metabolic reprogramming, to facilitate the clinical translation of targeted therapies. In summary, the dual prognostic and therapeutic value of PPAP2C offers a new breakthrough for precision medicine in breast cancer.

## Data Availability

The original contributions presented in the study are included in the article/Supplementary Material, further inquiries can be directed to the corresponding authors.
