# Supplementary material for: Integrated multi-omics analysis and functional experiments reveals PPAP2C as a potential prognostic biomarker and therapeutic target in breast cancer
Source: Front Pharmacol. 2026 Aug 24;17:1884232. doi: 10.3389/fphar.2026.1884232 (PMC13547041; doi:10.3389/fphar.2026.1884232)
Supplement: Supplementary file 1 [file DataSheet1.pdf]

Supplementary table and figures

Supplementary Table 1. Primers for quantitative real-time PCR (qPCR)

Gene	Forward Primer (5'→3')	Reverse Primer (5'→3')
PPAP2A	GGGCAGAGACCATGTTTGACA	CTCGTTGGAAGGGGGTATGC
PPAP2B	ACCACCGAGGGTTTTACTGC	AAGAGGAAGCAGCCCACTTG
GAPDH	GGAGCGAGATCCCTCCAAAAT	GGCTGTTGTCATACTTCTCATGG
PPAP2C-1	TGGTGTACACAGACCGGCTC	AGTTGACCCGGCTCCAGTCG
PPAP2C-2	ACACCCGCGTGTCTGATTAC	GGGGTCGGGCTTTGAAGAA

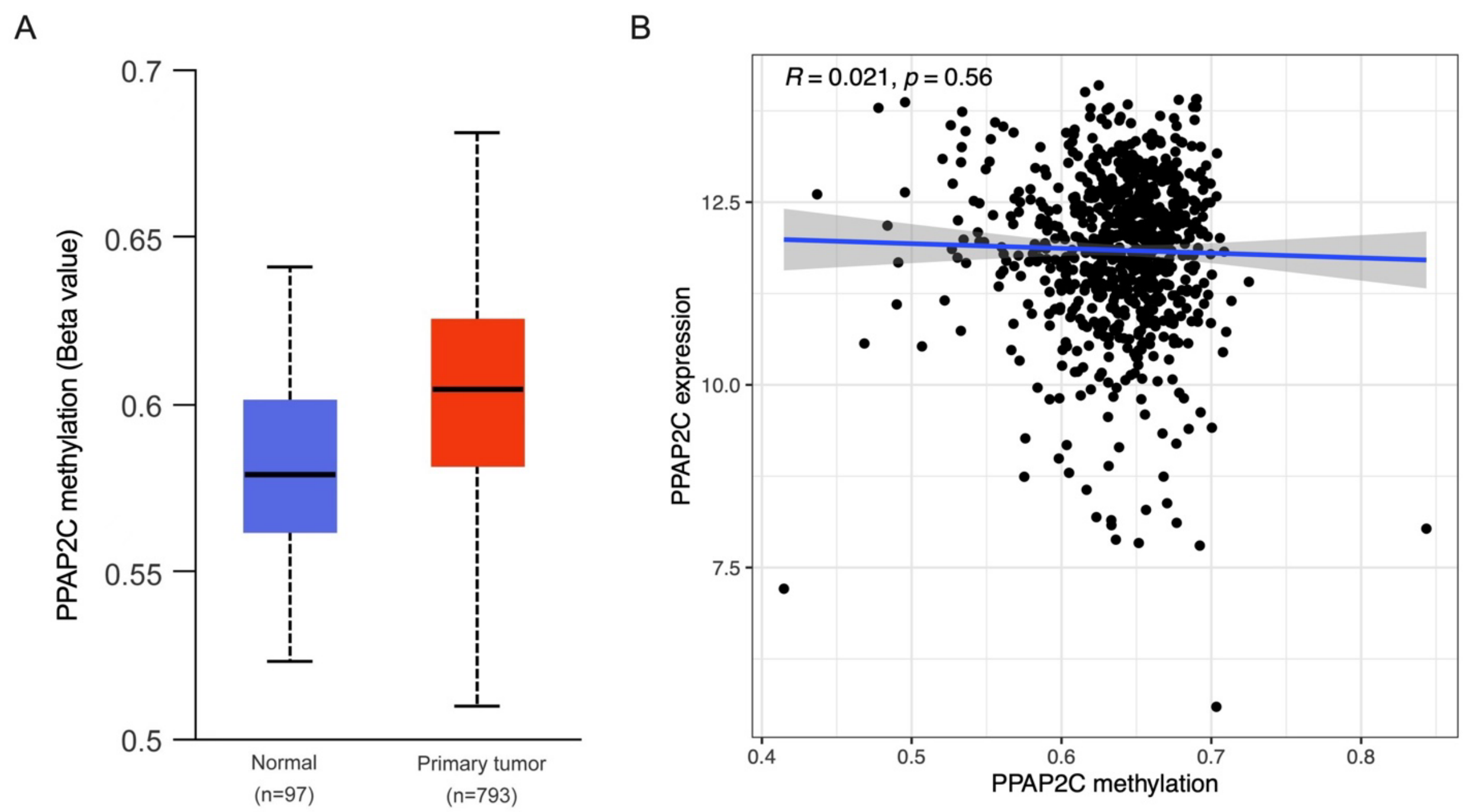

Supplementary Figure 1. Correlation between PPAP2C expression and its promoter methylation in breast cancer. (A) Boxplot showing PPAP2C promoter methylation beta values in normal breast tissues (n = 97) and primary tumor tissues (n = 793) from the TCGA breast cancer dataset. Data were obtained from the UALCAN database. The median beta value was 0.579 in normal tissues and 0.604 in tumor tissues. (B) Scatter plot showing the correlation between PPAP2C expression (log2(TPM+1)) and its promoter methylation beta values in TCGA breast cancer samples. Spearman's correlation analysis revealed no significant correlation between the two variables (R = 0.021, P = 0.56). TPM, transcripts per million

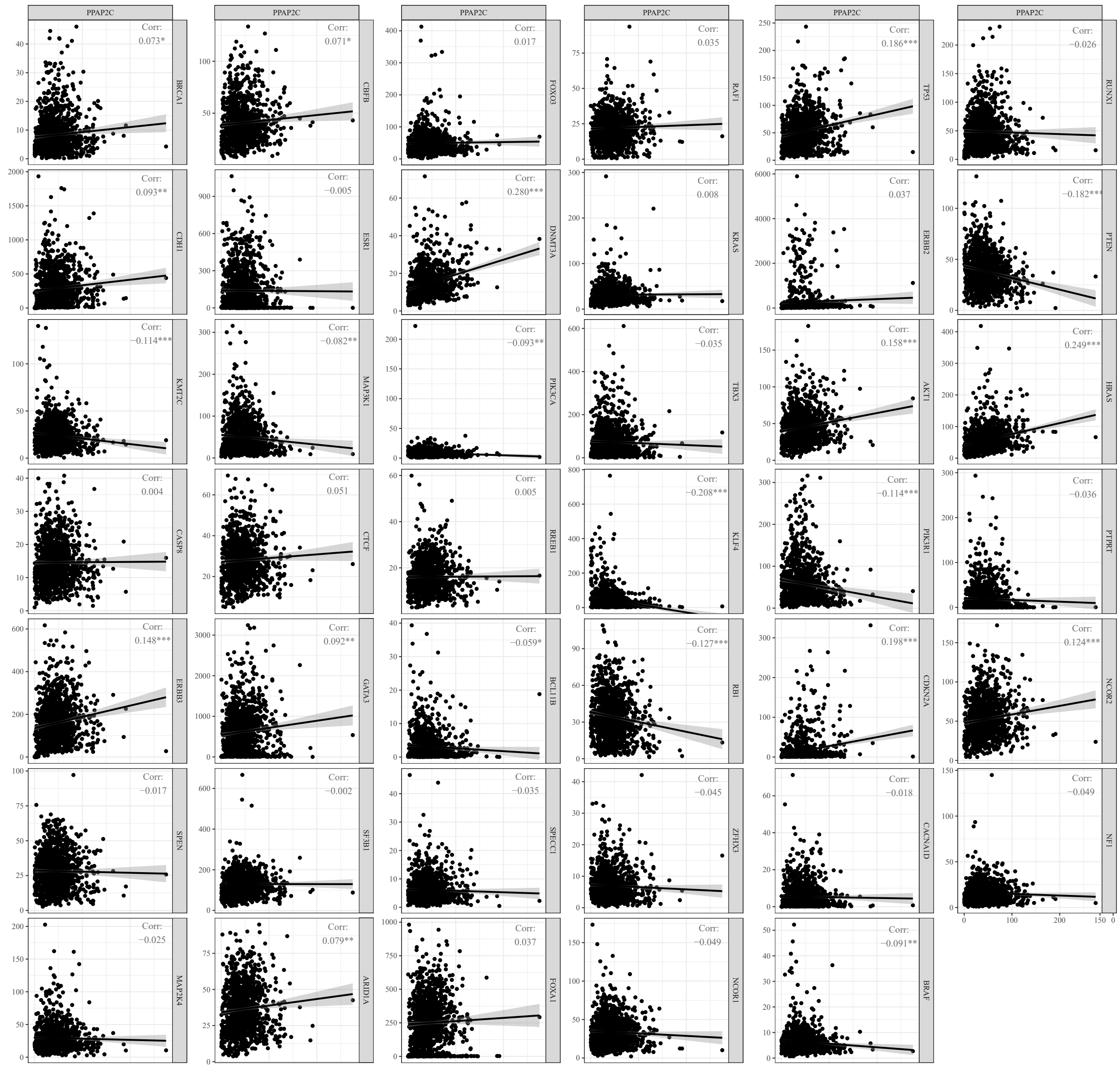

Supplementary Figure 2. Correlation between PPAP2C expression and 41 breast cancer driver genes. Scatter plots showing Spearman's correlations between PPAP2C expression and the expression levels of 41 known breast cancer driver genes in 1,217 TCGA breast cancer samples. Each panel represents the correlation between PPAP2C and one driver gene. Spearman's correlation coefficients (Corr) are shown in each panel, with significance indicated by asterisks (*$P < 0.05$, **$P < 0.01$, ***$P < 0.001$). None of the correlations reached the predefined significance threshold (|Corr| > 0.3).

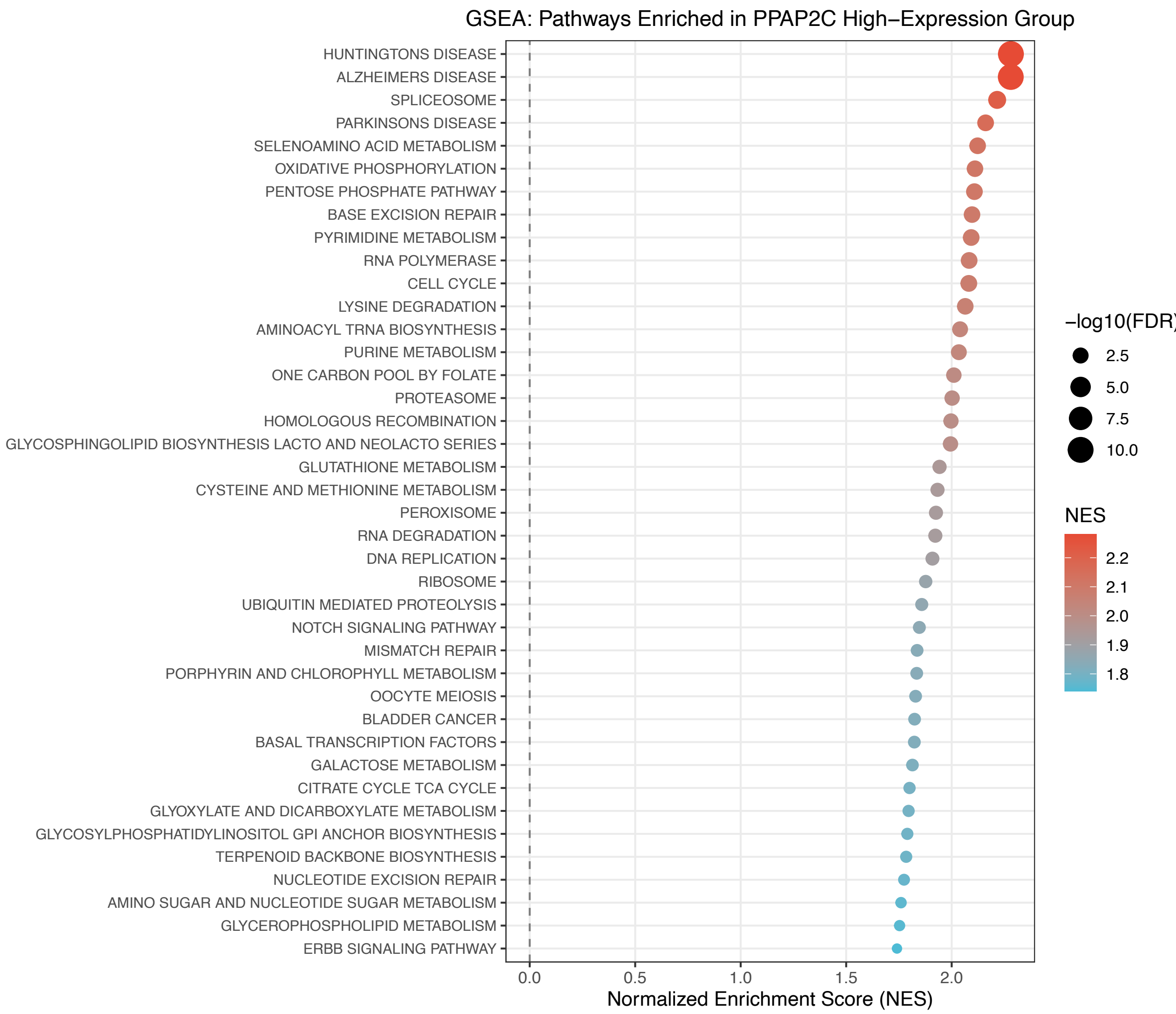

Supplementary Figure 3. Gene Set Enrichment Analysis of PPAP2C expression. Dot plot showing the 40 significantly enriched KEGG pathways in the PPAP2C high-expression group (FDR < 0.05). The size of each dot represents -log10(FDR), and the color gradient indicates the normalized enrichment score (NES). Pathways are ordered by NES. FDR, false discovery rate; NES, normalized enrichment score.

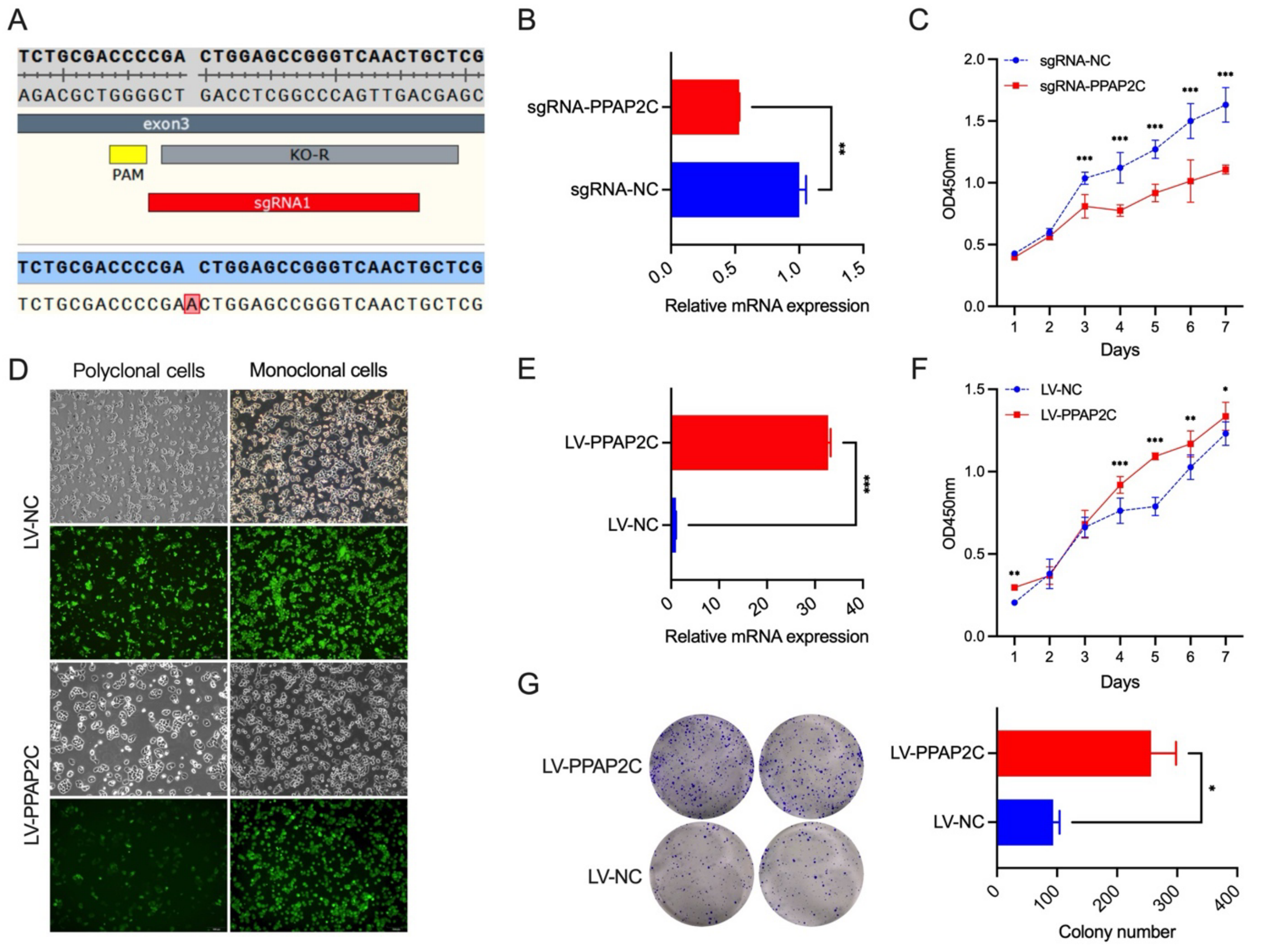

Supplementary Figure 4. PPAP2C regulates proliferative capacity and colony formation in MDA-MB-468 cells. (A) Schematic illustration of the CRISPR-Cas9-mediated knockout strategy for PPAP2C, accompanied by sequencing chromatograms confirming frameshift mutations in monoclonal cell lines. (B) Quantitative PCR (qPCR) analysis employing gene-specific primers to validate PPAP2C transcript knockdown. (C) CCK-8 proliferation assay monitoring growth dynamics of PPAP2C-knockout and control cells. (D) Representative bright-field and fluorescence (EGFP) images of PPAP2C-overexpressing cell lines established through lentiviral vector transduction and monoclonal selection. (E) Quantitative PCR (qPCR) analysis verifying PPAP2C mRNA overexpression in lentivirus-transduced and control cells. (F) CCK-8 proliferation assay monitoring growth dynamics of PPAP2C-overexpressing and control cells. (G) Colony formation assay comparing the clonogenicity between PPAP2C-overexpressing and control cells.

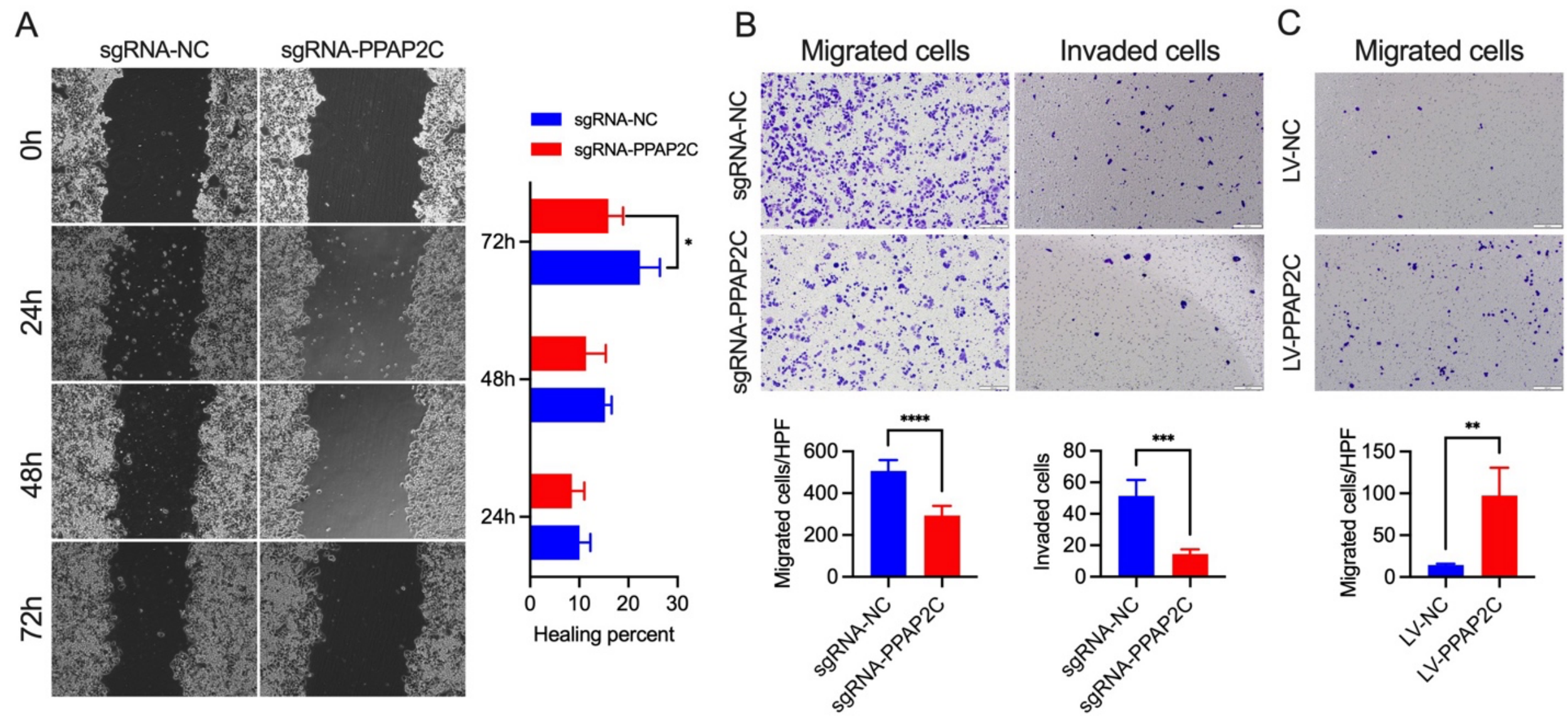

Supplementary Figure 5. PPAP2C promotes migration and invasion of MDA-MB-468 cells. (A) Wound healing assay comparing the migration capabilities of PPAP2C-knockout and control cells. (B) Transwell invasion assay evaluating the migration and invasion potentials of PPAP2C-knockout and control cells. (C) Transwell invasion assay evaluating the migration potentials of PPAP2C-overexpressing and control cells.

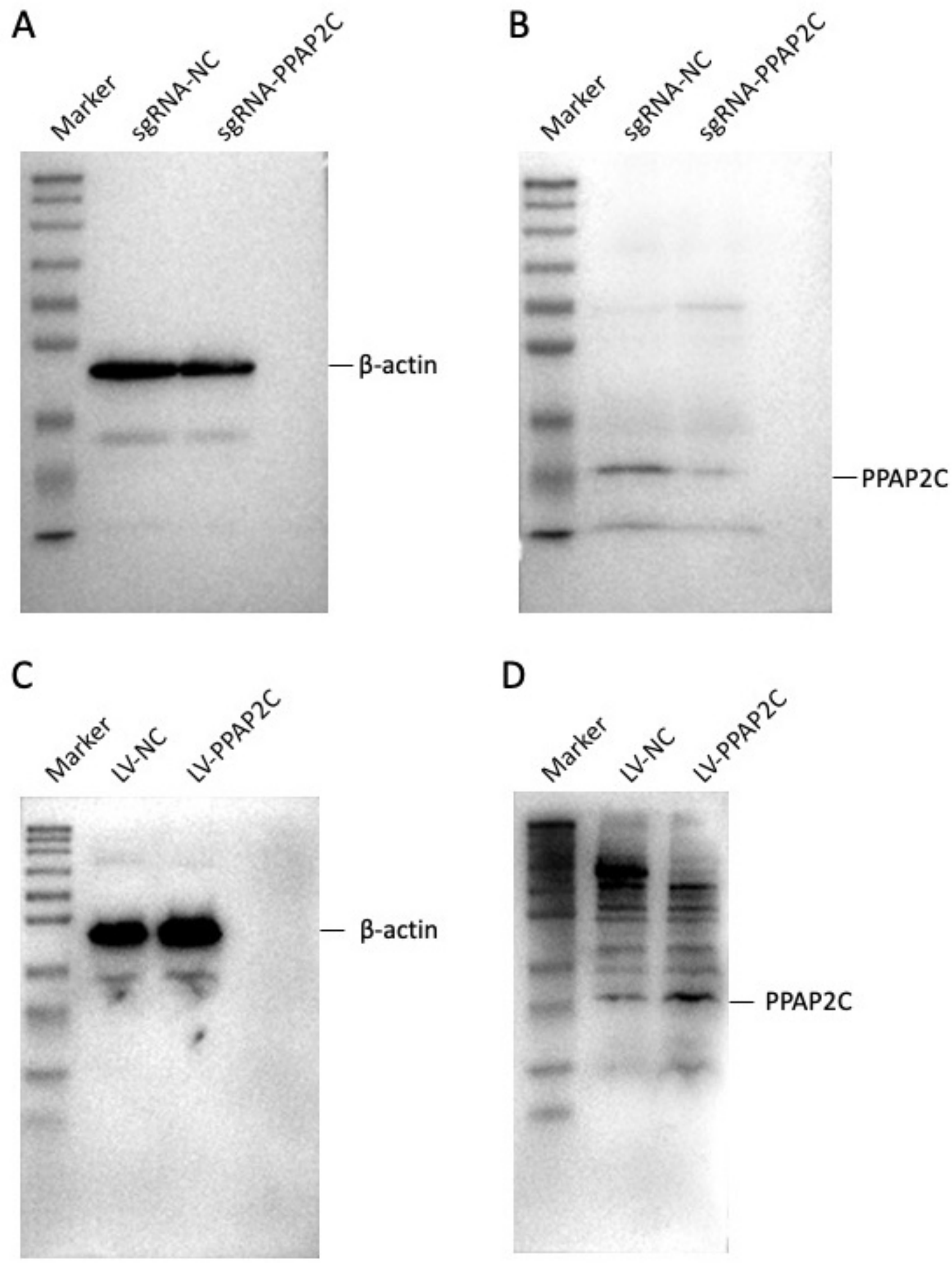

Supplementary Figure 6. Uncropped Western blot images corresponding to Figure 9C and 9G. (A) Detection of β-actin in control (sgRNA-NC) and PPAP2C-knockout (sgRNA-PPAP2C) MDA-MB-231 cells. (B) Detection of PPAP2C in control (sgRNA-NC) and PPAP2C-knockout (sgRNA-PPAP2C) MDA-MB-231 cells. (C) Detection of β-actin in MDA-MB-231 cells transduced with control (LV-NC) or PPAP2C-overexpressing (LV-PPAP2C) lentivirus. (D) Detection of PPAP2C in MDA-MB-231 cells transduced with control (LV-NC) or PPAP2C-overexpressing (LV-PPAP2C) lentivirus.
